# Risk of thrombocytopenia and thromboembolism after covid-19 vaccination and SARS-CoV-2 positive testing: self-controlled case series study

**DOI:** 10.1136/bmj.n1931

**Published:** 2021-08-26

**Authors:** Julia Hippisley-Cox, Martina Patone, Xue W Mei, Defne Saatci, Sharon Dixon, Kamlesh Khunti, Francesco Zaccardi, Peter Watkinson, Manu Shankar-Hari, James Doidge, David A Harrison, Simon J Griffin, Aziz Sheikh, Carol A C Coupland

**Affiliations:** 1 Nuffield Department of Primary Health Care Sciences, University of Oxford, Oxford, UK; 2 Leicester Real World Evidence Unit, Leicester Diabetes Centre, University of Leicester, Leicester, UK; 3 Nuffield Department of Clinical Neurosciences, University of Oxford, NIHR Biomedical Research Centre, Oxford University Hospitals NHS Trust, Oxford, UK; 4 Department of Critical Care Medicine, Guys and St Thomas’ NHS Foundation Trust, London, UK; 5 Intensive Care National Audit & Research Centre, London, UK; 6 Department of Medical Statistics, London School of Hygiene and Tropical Medicine, London, UK; 7 Primary Care Unit, Department of Public Health and Primary Care, School of Clinical Medicine, University of Cambridge, Cambridge, UK; 8 MRC Epidemiology Unit, Institute of Metabolic Science, School of Clinical Medicine, University of Cambridge, Cambridge, UK; 9 Usher Institute, University of Edinburgh, Edinburgh, UK; 10 Division of Primary Care, School of Medicine, University of Nottingham, Nottingham, UK

## Abstract

**Objective:**

To assess the association between covid-19 vaccines and risk of thrombocytopenia and thromboembolic events in England among adults.

**Design:**

Self-controlled case series study using national data on covid-19 vaccination and hospital admissions.

**Setting:**

Patient level data were obtained for approximately 30 million people vaccinated in England between 1 December 2020 and 24 April 2021. Electronic health records were linked with death data from the Office for National Statistics, SARS-CoV-2 positive test data, and hospital admission data from the United Kingdom’s health service (NHS).

**Participants:**

29 121 633 people were vaccinated with first doses (19 608 008 with Oxford-AstraZeneca (ChAdOx1 nCoV-19) and 9 513 625 with Pfizer-BioNTech (BNT162b2 mRNA)) and 1 758 095 people had a positive SARS-CoV-2 test. People aged ≥16 years who had first doses of the ChAdOx1 nCoV-19 or BNT162b2 mRNA vaccines and any outcome of interest were included in the study.

**Main outcome measures:**

The primary outcomes were hospital admission or death associated with thrombocytopenia, venous thromboembolism, and arterial thromboembolism within 28 days of three exposures: first dose of the ChAdOx1 nCoV-19 vaccine; first dose of the BNT162b2 mRNA vaccine; and a SARS-CoV-2 positive test. Secondary outcomes were subsets of the primary outcomes: cerebral venous sinus thrombosis (CVST), ischaemic stroke, myocardial infarction, and other rare arterial thrombotic events.

**Results:**

The study found increased risk of thrombocytopenia after ChAdOx1 nCoV-19 vaccination (incidence rate ratio 1.33, 95% confidence interval 1.19 to 1.47 at 8-14 days) and after a positive SARS-CoV-2 test (5.27, 4.34 to 6.40 at 8-14 days); increased risk of venous thromboembolism after ChAdOx1 nCoV-19 vaccination (1.10, 1.02 to 1.18 at 8-14 days) and after SARS-CoV-2 infection (13.86, 12.76 to 15.05 at 8-14 days); and increased risk of arterial thromboembolism after BNT162b2 mRNA vaccination (1.06, 1.01 to 1.10 at 15-21 days) and after SARS-CoV-2 infection (2.02, 1.82 to 2.24 at 15-21 days). Secondary analyses found increased risk of CVST after ChAdOx1 nCoV-19 vaccination (4.01, 2.08 to 7.71 at 8-14 days), after BNT162b2 mRNA vaccination (3.58, 1.39 to 9.27 at 15-21 days), and after a positive SARS-CoV-2 test; increased risk of ischaemic stroke after BNT162b2 mRNA vaccination (1.12, 1.04 to 1.20 at 15-21 days) and after a positive SARS-CoV-2 test; and increased risk of other rare arterial thrombotic events after ChAdOx1 nCoV-19 vaccination (1.21, 1.02 to 1.43 at 8-14 days) and after a positive SARS-CoV-2 test.

**Conclusion:**

Increased risks of haematological and vascular events that led to hospital admission or death were observed for short time intervals after first doses of the ChAdOx1 nCoV-19 and BNT162b2 mRNA vaccines. The risks of most of these events were substantially higher and more prolonged after SARS-CoV-2 infection than after vaccination in the same population.

**Figure:**
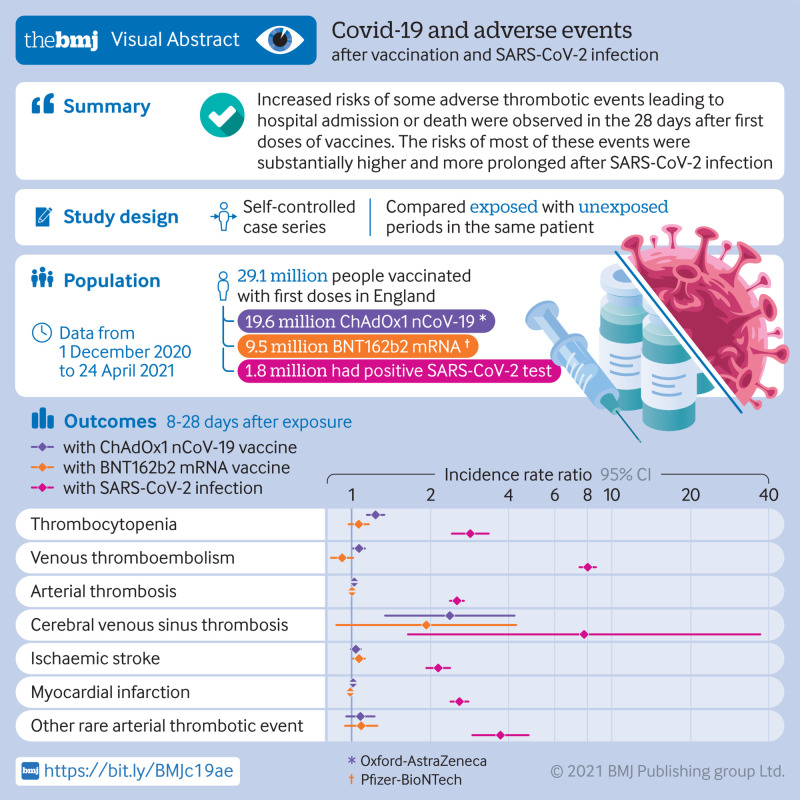


## Introduction

Covid-19 vaccines have been tested in randomised clinical trials^1^ ^2^ ^3^ that are designed to establish efficacy and safety, but insufficiently powered to detect rare adverse events. If a new vaccine has a serious adverse profile (even if the risk is rare), then a risk-benefit evaluation could lead to withdrawal of the vaccine or restrictions on its use in the interests of public safety. These risks, however, need to be balanced against the risks of covid-19 itself leading to adverse events and death.^4^ Safety evaluation is particularly important when vaccine development has been accelerated, the vaccines are new, and when rapid worldwide deployment of covid-19 vaccines has occurred, with 1.43 billion doses already administered worldwide.^5^

Recently, thrombocytopenia and rare thromboembolic events after the ChAdOx1 nCoV-19 (Oxford-AstraZeneca) vaccine have been reported in Denmark, Norway,^6^ ^7^ Germany, Austria,^8^ and the United Kingdom.^9^ This prompted several countries to limit the use of ChAdOx1 nCoV-19 on the basis that the benefits might not outweigh the risks.^8^ ^9^ ^10^ ^11^ The United States has reported similar events associated with the Ad26.COV2-S (recombinant) Janssen vaccine, resulting in a pause in its roll-out.^12^

The situation is further complicated by evidence on the associations between SARS-CoV-2 infection and thromboses.^4^ A US study estimated that the risk of cerebral venous sinus thrombosis (CVST) associated with SARS-CoV-2 infection is approximately seven times greater than that associated with the BNT162b2 mRNA (Pfizer-BioNTech) or mRNA-1273 (Moderna) covid-19 vaccines.^13^ Direct comparisons between these vaccines and the ChAdOx1 nCoV-19 vaccine were not possible because the ChAdOx1 nCoV-19 vaccine is not used in the US. Evaluation of the safety of new vaccines relies on case reports or series, and analyses of large scale health data. While informative, case reports are limited by small numbers, and potential selection, recall, and recording biases. In contrast, routinely collected electronic health data contain recording of the exposure, outcomes, and confounders, and provide a rich source of data to enable the robust evaluation of rare vaccine safety events.^14^

We evaluated the short term risks of thrombocytopenia, venous thromboembolism, and arterial thromboembolism associated with the first dose of the ChAdOx1 nCoV-19 and BNT162b2 vaccines, or a SARS-CoV-2 positive test in England between 1 December 2020 and 24 April 2021. We also evaluated the risk of prespecified secondary outcomes of interest, namely CVST, ischaemic stroke, myocardial infarction, and other rare arterial thrombotic events according to a prespecified protocol.^15^

## Methods

### Study design and period

We undertook a self-controlled case series from 1 December 2020 to 24 April 2021 (the latest date for which outcome data were available) to examine the associations between ChAdOx1 nCoV-19 or BNT162b2 mRNA vaccines and thrombotic events during the ongoing covid-19 vaccination programme in England. We also investigated the association between a SARS-CoV-2 positive test and the thrombotic events of interest among the same vaccinated population.

The self-controlled case series was originally developed to assess risks of adverse events to vaccination.^16^ The case series determines the relative incidence of the outcome of interest for exposed time periods (eg, after vaccination or SARS-CoV-2 infection) compared with unexposed baseline periods in people with the outcome of interest (see supplementary fig 1). Inference is within people and therefore this implicitly controls for all covariates that remain constant during the study period. We selected patients with each outcome during the study period and determined dates when they had the vaccine or tested positive for SARS-CoV-2. Separate analyses were carried out for each outcome of interest.

### Data sources

We used the National Immunisation Management System register of covid-19 vaccination to identify vaccine exposure, which includes vaccine type, date, and doses for all people vaccinated in England. We linked individual level data to national data for mortality, hospital admissions, and SARS-CoV-2 infection. Linkages are conducted to protect patient anonymity because the hashed ID used to link patients cannot be reversed. Vaccine and SARS-CoV-2 positive test data were available until 24 April 2021. Hospital admission data were obtained from two sources: Hospital Episode Statistics (up to 31 March 2021), which includes any admission, and Secondary Users Service (up to 24 April 2021), which includes only admissions with an outcome (death or discharge).

We used the QResearch database of 12 million patients linked to the above dataset to calculate background incidence rates for each outcome before the pandemic (2015-19). QResearch includes demographic, clinical, and drug data, and is used for clinical^17^ ^18^ and drug safety research.^19^ ^20^ QResearch is one of the largest and most representative primary care research databases nationally,^21^ covering approximately 20% of the population of England.

### Inclusion criteria

We included all people aged ≥16 years who had first doses of the ChAdOx1 nCoV-19 or BNT162b2 mRNA vaccines and any outcome of interest. We excluded people who received the mRNA-1273 (Moderna) vaccine because of very small numbers. We used the first event in the study period and excluded patients from the analysis of each outcome if they had a hospital admission for the same event in the two years before the start of the study period.

### Exposure

Our main exposures were the first dose of the ChAdOx1 nCoV-19 or the BNT162b2 mRNA vaccine. We censored people on the earliest of the following: date of their second dose, date of death, or study end date (24 April 2021). Our secondary exposure was a positive SARS-CoV-2 test result, using the first date of a positive test (not the date of reporting) as the exposure date. We defined the exposure risk intervals as the following prespecified time periods: 0, 1-7, 8-14, 15-21, and 22-28 days after each exposure date (vaccination date or date of positive SARS-CoV-2 test). Multiple risk intervals were used to differentiate between acute and non-acute phases after vaccination. The baseline period (without exposure) was defined as any time between 1 December 2020 and 29 days before the exposure date, and from 29 days after the exposure date until 24 April 2021 or the censored date if earlier. A pre-risk interval of 1-28 days before each exposure date was included to deal with possible bias that might arise if the occurrence of the outcome temporarily influenced the likelihood of exposure.^22^ Histograms of the data by interval between vaccination and event day are shown in supplementary figure 2a. The graphs show a drop of hospital admissions or deaths for each outcome about 28 days before vaccination, hence the choice of the pre-risk period. Hospital admissions for the event of interest are probably the trigger for the covid-19 test. Such events might be caused by SARS-CoV-2 infection, but the reverse causality involved in their detection induces bias. To reduce the bias, which could overestimate or underestimate the effect of infection, we decided to allocate day 0 to a risk period on its own.^23^

### Outcomes

Our three composite primary outcomes were hospital admission or death associated with thrombocytopenia, venous thromboembolism, and arterial thromboembolism. Our prespecified secondary outcomes were subsets of the primary outcomes: CVST, ischaemic stroke, myocardial infarction, and other rare arterial thrombotic events. Hospital inpatient admissions from 1 December 2020 to 24 April 2021 with an ICD-10 (International Classification of Diseases version 10) diagnosis code in their first 13 diagnoses fields that indicated the outcome of interest were identified in the Hospital Episode Statistics and Secondary Users Service databases. Supplementary table 1 gives a list of ICD-10 codes and groupings for each outcome. We used the earliest date of hospital admission or date of death for the relevant event as the event date. Cause of death in the UK is assessed by the medical practitioner who attended the patient during their last illness, and if not seen in the preceding 14 days, the cause is determined by a coroner based on assessment of medical evidence.

### Statistical analysis

We described the characteristics of each cohort (patients who had been vaccinated with the outcomes of interest) in terms of age, sex, and ethnicity. The self-controlled case series models were fitted using a conditional Poisson regression model with an offset for the length of the risk period. Incidence rate ratios, the relative rate of hospital admissions or deaths due to each outcome of interest in risk periods relative to baseline periods, and their 95% confidence intervals were estimated using each model. Exposure terms for both vaccines and for infection with SARS-CoV-2 were included in the same model. To account for temporal changes in background rates, we divided the study period into weekly blocks starting on 1 December 2020 and adjusted for these changes as discrete covariates in the analysis. We used Wald tests to compare risks associated with ChAdOx1 nCoV-19 and BNT162b2 mRNA vaccines. We investigated whether the associations between vaccine exposures and outcomes are sex or age dependent by running the analyses in separate subgroups by sex and age group (younger or older than 50 years).

### Sensitivity analyses

We conducted six sensitivity analyses: (1) excluding those who died from the outcome; (2) restricting analysis to the period after vaccination or after a SARS-CoV-2 positive test, without censoring at death; (3) censoring at 12 weeks after vaccination; (4) censoring on 10 March 2021; (5) restricting the study period until 31 March 2021; and (6) restricting the analysis to patients who had their vaccination after 1 January 2021. The first two analyses tested the assumption that the occurrence of an outcome event did not influence the probability of subsequent exposures, such as through death.^22^ The third analysis tested the sensitivity to our approach of censoring patients at the time of the second dose (to avoid contamination of baseline time). The additional censoring at 12 weeks after the first dose of vaccine was used because this is the recommended time for a second dose in the UK. Concerns over CVST and blood clots were raised first in Europe around 10 March 2021, and so we censored on this date for the fourth sensitivity analysis; this allowed us to include only time unaffected by any notoriety bias (because of media attention, thrombotic events might have been more likely to be reported on death certificates or hospital admission records if healthcare professionals were aware of recent ChAdOx1 nCoV-19 vaccination). The fifth analysis tested the completeness of the data, and the sixth analysis was done to have comparable time periods and priority groups for the two vaccines.

### Assessing the self-controlled case series assumptions

To further assess the assumptions of the self-controlled case series and our modelling choices, we visually examined the data. We plotted a histogram of the number of occurrences of an event by time before or since vaccination for each outcome to assess the possibility that a hospital admission for that event might affect subsequent vaccination (supplementary fig 2a). We plotted a histogram of the time from event to actual end of observation in patients who were censored and uncensored to assess if event dependent observation periods could be a problem for the analysis (supplementary fig 2b).

*Event dependent exposures*—supplementary figure 2a shows the number of occurrences of an event by time before or after vaccination. We observed a decrease in the 28 days immediately before vaccination, indicating that occurrence of an event might have reduced the likelihood of vaccination. This pattern is similar for most of the outcomes and for both vaccines. Therefore, we have added the pre-risk period of 28 days.

*Event dependent observation periods*—supplementary figure 2b shows the frequency of days from event to actual end of observation in censored and uncensored patients. A spike close to zero is apparent in the censored data histogram for most of the outcomes, excluding CVST. This finding indicates the presence of event dependent observation periods (censoring on death date due to outcome), which we tested further with the first and second sensitivity analyses (excluding those who died from the outcome; restricting analysis to the period after vaccination, without censoring at death). These additional analyses agreed with the main analysis, suggesting that there should be little concern about these outcomes.

### Absolute measure of risk

In self-controlled case series analysis, results are presented in relative terms—the ratio of the incidence in the exposure risk periods relative to the incidence in control periods. We supplemented these results with measures of effect of each exposure in absolute terms using a method developed to estimate the number of exposures needed to produce one excess adverse outcome and the excess number of events per 10 million exposed for each outcome.^24^ These measures were computed for a period of 8-28 days after vaccination to remove the healthy vaccinee effect seen at 0-7 days after vaccination. To make numbers comparable, the same measures were computed for the same period after a SARS-CoV-2 positive test. 

### Negative or positive controls

We examined the associations of exposures with coeliac disease as a negative control outcome^25^ because it was not thought to be associated with exposure to vaccination or SARS-CoV-2 infection; and with anaphylaxis as a positive control outcome because it could occur shortly after either vaccine.^26^

### Patient and public involvement

This project is supported by a patient and public involvement advisory panel which we thank for its continued support and guidance. The input of the panel has helped us identify priority questions for consideration. PPIE (patient and public involvement and engagement) advisers were supportive of the vital importance of reporting on thrombosis risks associated with vaccination against covid-19 and covid-19 itself. We have reviewed the findings of this study with our PPIE advisers. A lay summary has been developed with patient and public involvement input and feedback, including an infographic. 

## Results

Table 1 shows the characteristics of 19 608 008 people who had the ChAdOx1 nCoV-19 vaccine, 9 513 625 who had the BNT162b2 mRNA vaccine, and 1 758 095 with a SARS-CoV-2 positive test. During the study period, among those vaccinated, 9764 people had a hospital admission related to thrombocytopenia (52 deaths) and 23 390 people were admitted to hospital with venous thromboembolism (1871 deaths); this included 119 people with CVST related hospital admissions (no deaths). Hospital admission related to arterial thromboembolic events occurred in 89 321 people (6533 deaths); these included 28 222 ischaemic strokes (4204 deaths), 62 699 with myocardial infarction (2875 deaths), and 3655 with other rare arterial thrombotic events (84 deaths). Table 2 shows the demographic characteristics of patients who experienced the primary outcomes of interest in the 28 days after exposure. Supplementary table 2 shows corresponding results for the secondary outcomes.

**Table 1:** Baseline demographic characteristics of people receiving first dose of covid-19 vaccine or testing positive for SARS-CoV-2 virus among vaccinated population in England from 1 December 2020 to 24 April 2021. Figures are column % (number) unless stated otherwise

Characteristics	ChAdOx1 nCoV-19 vaccine	BNT162b2 mRNA vaccine	Positive SARS-CoV-2 test (among vaccinated population)
Total No of people	19 608 008	9 513 625	1 758 095
Sex			
Women	50.1 (9 822 236)	57.2 (5 443 631)	56.7 (996 462)
Men	45.1 (8 848 683)	40.0 (3 808 484)	40.0 (703 381)
Not recorded	4.8 (937 088)	2.7 (261 510)	3.3 (58 252)
Mean age, years (SD)	55.5 (14.9)	61.5 (18.8)	51.7 (17.0)
Age group, years			
16-29	5.4 (1 060 982)	7.2 (685 100)	10.9 (191 741)
30-39	7.9 (1 551 528)	8.3 (786 815)	12.6 (220 712)
40-49	19.4 (3 803 176)	10.8 (1 030 833)	20.9 (367 869)
50-59	28.4 (5 564 739)	15.6 (1 486 062)	26.7 (469 206)
60-69	20.3 (3 988 397)	17.8 (1 692 935)	14.4 (252 849)
70-79	14.2 (2 782 590)	20.3 (1 934 771)	7.2 (126 296)
80-89	3.3 (643 058)	17.0 (1 619 781)	5.1 (90 261)
≥90	1.1 (213 537)	2.9 (277 328)	2.2 (39 161)
Ethnicity			
White	72.9 (14 298 075)	77.4 (7 367 723)	71.3 (1 253 446)
Indian	2.2 (425 195)	2.5 (239 326)	3.9 (69 356)
Pakistani	1.3 (256 270)	1.0 (97 066)	2.8 (49 689)
Bangladeshi	0.6 (109 817)	0.4 (36 488)	1.1 (19 698)
Other Asian	1.0 (192 997)	1.1 (102 139)	1.7 (30 082)
Black Caribbean	0.6 (120 296)	0.6 (53 076)	0.7 (12 732)
Black African	1.1 (213 931)	1.0 (91 527)	1.6 (28 592)
Chinese	0.4 (69 563)	0.3 (26 228)	0.2 (3553)
Other ethnic group	1.9 (364 117)	1.7 (157 233)	2.5 (43 879)
Ethnicity not recorded	18.1 (3 557 746)	14.1 (1 342 819)	14.1 (247 068)
Previous conditions			
Previous thrombocytopenia	0.1 (25 917)	0.2 (18 004)	0.3 (4543)
Previous arterial thromboembolism	1.5 (294 698)	2.7 (254 295)	2.4 (41 821)
Myocardial infarction	1.2 (227 594)	2.1 (201 610)	1.8 (31 566)
Ischaemic stroke	0.3 (68 124)	0.6 (53 982)	0.6 (10 783)
Other rare arterial thrombosis	0.0 (9671)	0.1 (6727)	0.1 (1515)
Previous venous thromboembolism	0.2 (42 247)	0.3 (29 173)	0.5 (8671)
Previous CVST	0.0 (280)	0.0 (155)	0.0 (30)

BNT162b2 mRNA=Pfizer-BioNTech; CVST=cerebral venous sinus thrombosis; ChAdOx1 nCoV-19=Oxford-AstraZeneca vaccine.

**Table 2:** Demographic characteristics of patients who experienced outcome in 28 days after first dose of covid-19 vaccine or SARS-CoV-2 infection among vaccinated population in England from 1 December 2020 to 24 April 2021. Figures are column % (number) unless stated otherwise

Characteristics	Thrombocytopenia				Venous thromboembolism				Arterial thrombosis		
	ChAdOx1 nCoV-19 vaccine	BNT162b2 mRNA vaccine	Positive SARS-CoV-2 test		ChAdOx1 nCoV-19 vaccine	BNT162b2 mRNA vaccine	Positive SARS-CoV-2 test		ChAdOx1 nCoV-19 vaccine	BNT162b2 mRNA vaccine	Positive SARS-CoV-2 test
Total No of people with event	1480	1010	950		3077	2054	4671		11 617	9473	4076
Sex											
Women	47.8 (707)	43.5 (439)	39.5 (375)		52.2 (1605)	50.7 (1041)	38.4 (1795)		39.3 (4563)	39.3 (3719)	34.8 (1418)
Men	52.2 (773)	56.5 (571)	60.5 (575)		47.5 (1462)	49.2 (1011)	61.6 (2876)		60.6 (7044)	60.7 (5749)	65.2 (2658)
Not recorded	0	0	0		0.3 (10)	0.1 (<5)	0		0.1 (10)	0.1 (5)	0
Mean age (SD)	66.3 (16.0)	73.1 (15.5)	67.9 (16.0)		68.8 (14.7)	73.5 (13.8)	63.8 (14.8)		72.2 (12.5)	76.7 (11.7)	72.2 (13.5)
Age group, years											
16-29	2.5 (37)	1.7 (17)	1.8 (17)		1.8 (54)	1.1 (22)	1.3 (60)		0.1 (14)	0.1 (5)	*
30-39	4.7 (69)	3.0 (30)	4.0 (38)		2.8 (87)	1.5 (30)	4.3 (199)		0.6 (66)	0.3 (32)	<30
40-49	7.7 (114)	4.7 (47)	7.2 (68)		5.0 (153)	3.7 (77)	10.3 (482)		2.7 (314)	1.6 (155)	4.5 (182)
50-59	15.3 (227)	7.6 (77)	16.7 (159)		13.8 (424)	9.7 (199)	24.2 (1131)		13.0 (1512)	7.7 (729)	14.3 (583)
60-69	22.9 (339)	15.0 (152)	20.3 (193)		22.4 (689)	15.1 (310)	23.6 (1102)		23.1 (2680)	15.2 (1437)	21.7 (886)
70-79	26.1 (387)	24.6 (248)	22.4 (213)		32.1 (987)	28.7 (590)	20.5 (959)		33.3 (3871)	26.5 (2507)	24.8 (1010)
80-89	14.9 (220)	35.6 (360)	21.1 (200)		16.3 (501)	33.0 (677)	12.4 (580)		18.0 (2090)	37.9 (3592)	24.3 (992)
≥90	5.9 (87)	7.8 (79)	6.5 (62)		5.9 (182)	7.3 (149)	3.4 (158)		9.2 (1070)	10.7 (1016)	9.7 (394)
Ethnicity											
White	89.3 (1321)	89.3 (902)	83.2 (790)		90.3 (2777)	89.9 (1847)	76.2 (3558)		88.7 (10 310)	89.5 (8483)	80.4 (3279)
Indian	2.4 (35)	1.8 (18)	2.9 (28)		1.2 (38)	1.3 (27)	3.5 (163)		2.4 (279)	2.5 (233)	4.3 (174)
Pakistani	0.9 (13)	1.2 (12)	3.1 (29)		0.6 (17)	0.7 (15)	2.4 (113)		1.6 (184)	1.3 (121)	4.0 (163)
Bangladeshi	0.5 (7)	<10	1.3 (12)		0.3 (8)	*	1.3 (59)		0.4 (46)	0.3 (29)	1.4 (59)
Other Asian	0.8 (12)	1.0 (10)	1.6 (15)		0.3 (8)	0.8 (16)	1.8 (84)		0.6 (71)	0.6 (55)	1.7 (69)
Black Caribbean	0.9 (13)	0.6 (6)	1.1 (10)		1.3 (39)	0.8 (16)	2.6 (121)		0.7 (79)	0.5 (49)	1.1 (43)
Black African	0.9 (13)	0.5 (5)	1.6 (15)		0.7 (23)	1.0 (20)	3.1 (143)		0.5 (60)	0.4 (40)	1.0 (41)
Chinese	*	*	*		*	*	0.2 (10)		0.1 (16)	0.1 (13)	0.3 (12)
Other ethnic group	1.2 (18)	1.2 (12)	2.2 (21)		1.0 (32)	1.0 (21)	3.4 (158)		1.1 (122)	1.0 (93)	2.5 (103)
Ethnicity not recorded	3.2 (47)	3.7 (37)	2.9 (28)		4.3 (132)	4.3 (88)	5.6 (262)		3.9 (450)	3.8 (357)	3.3 (133)

BNT162b2 mRNA=Pfizer-BioNTech vaccine; ChAdOx1 nCoV-19=Oxford-AstraZeneca vaccine.

* Cells with numbers <5 are suppressed.

**Fig 1:**
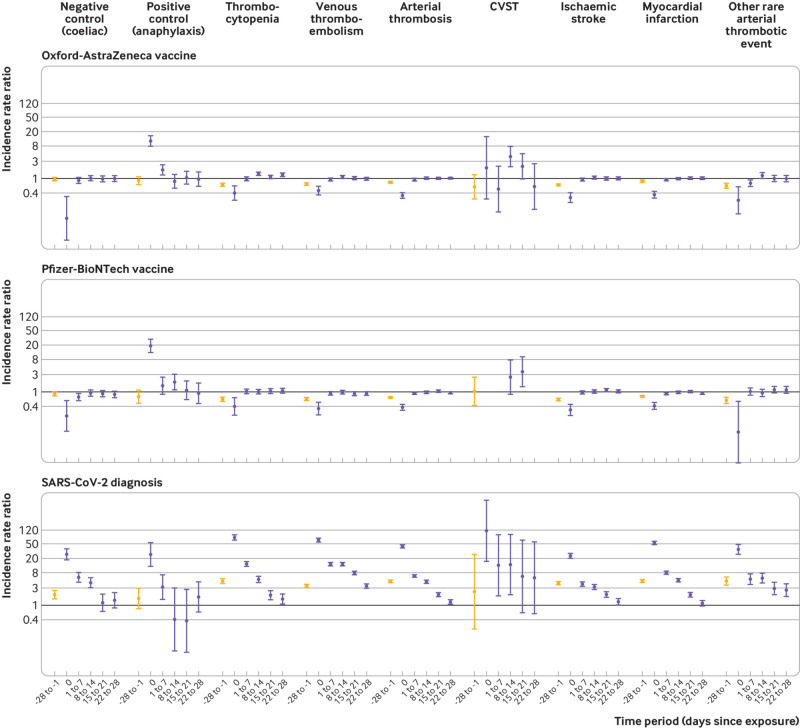
Incidence rate ratios (95% confidence intervals) for outcomes in predefined risk periods immediately before and after exposure to ChAdOx1 nCoV-19 (Oxford-AstraZeneca) or BNT162b2 mRNA (Pfizer-BioNTech) vaccine, and before and after a positive SARS-CoV-2 test result from 1 December 2020 to 24 April 2021. CVST=cerebral venous sinus thrombosis

Table 3 and figure 1 show the number of patients with outcome events in each time period and the incidence rate ratios for outcomes in the risk periods immediately before and after each exposure.

**Table 3:** Incidence rate ratios (95% confidence intervals) for primary composite and secondary outcomes in predefined risk periods immediately before and after exposure to vaccine and before and after positive SARS-CoV-2 test result, adjusted for calendar time from 1 December 2020 to 24 April 2021

Outcome and time period	ChAdOx1 nCoV-19 vaccine			BNT162b2 mRNA vaccine			Positive SARS-CoV-2 test	
	No of events	Incidence rate ratio (95% CI)		No of events	Incidence rate ratio (95% CI)		No of events	Incidence rate ratio (95% CI)
**Composite primary outcomes**								
Thrombocytopenia								
Baseline	3851	1.00		2009	1.00		381	1.00
−28 to −1 days	910	0.67 (0.62 to 0.72)		504	0.63 (0.56 to 0.69)		430	4.71 (4.06 to 5.47)
0 day	19	0.39 (0.25 to 0.62)		13	0.41 (0.23 to 0.70)		299	75.77 (64.48 to 89.03)
1-7 days	331	0.97 (0.87 to 1.10)		243	1.02 (0.89 to 1.18)		398	14.04 (12.08 to 16.31)
8-14 days	438	1.33 (1.19 to 1.47)		254	1.02 (0.89 to 1.17)		152	5.27 (4.34 to 6.40)
15-21 days	337	1.08 (0.96 to 1.22)		259	1.06 (0.93 to 1.22)		56	1.91 (1.44 to 2.54)
22-28 days	356	1.26 (1.13 to 1.42)		241	1.08 (0.94 to 1.23)		45	1.50 (1.10 to 2.05)
Venous thromboembolism								
Baseline	9846	1.00		4627	1.00		1211	1.00
−28 to −1 days	2561	0.69 (0.66 to 0.73)		1224	0.64 (0.60 to 0.68)		1007	3.43 (3.14 to 3.76)
0 day	53	0.46 (0.35 to 0.60)		25	0.35 (0.24 to 0.52)		833	63.52 (57.80 to 69.80)
1-7 days	746	0.92 (0.85 to 1.00)		486	0.92 (0.83 to 1.01)		1305	13.78 (12.66 to 14.99)
8-14 days	870	1.10 (1.02 to 1.18)		555	0.99 (0.90 to 1.08)		1371	13.86 (12.76 to 15.05)
15-21 days	763	1.03 (0.96 to 1.12)		514	0.91 (0.82 to 1.00)		807	7.88 (7.18 to 8.64)
22-28 days	645	0.97 (0.89 to 1.06)		474	0.89 (0.81 to 0.99)		355	3.38 (3.00 to 3.81)
Arterial thromboembolism								
Baseline	31 944	1.00		21 147	1.00		2743	1.00
−28 to −1 days	9155	0.78 (0.76 to 0.80)		5984	0.71 (0.69 to 0.73)		3054	4.59 (4.34 to 4.86)
0 day	143	0.33 (0.28 to 0.39)		125	0.38 (0.32 to 0.45)		1165	42.26 (39.33 to 45.40)
1-7 days	2769	0.92 (0.88 to 0.95)		2161	0.92 (0.88 to 0.96)		1290	6.55 (6.12 to 7.02)
8-14 days	3065	1.02 (0.98 to 1.06)		2377	0.98 (0.94 to 1.02)		917	4.52 (4.19 to 4.88)
15-21 days	2906	1.01 (0.97 to 1.05)		2590	1.06 (1.01 to 1.10)		426	2.02 (1.82 to 2.24)
22-28 days	2734	1.02 (0.98 to 1.06)		2221	0.95 (0.91 to 0.99)		278	1.26 (1.11 to 1.43)
**Secondary outcomes**								
Cerebral venous sinus thrombosis								
Baseline	44	1.00		21	1.00		*	1.00
−28 to −1 days	9	0.59 (0.27 to 1.25)		7	1.04 (0.43 to 2.51)		*	2.40 (0.22 to 25.98)
0 day	*	1.95 (0.26 to 14.48)		*	NA		*	115.70 (16.48 to 812.29)
1-7 days	*	0.51 (0.12 to 2.19)		*	NA		*	12.90 (1.86 to 89.64)
8-14 days	15	4.01 (2.08 to 7.71)		*	2.57 (0.85 to 7.78)		*	13.43 (1.99 to 90.59)
15-21 days	8	2.15 (0.96 to 4.85)		6	3.58 (1.39 to 9.27)		*	6.33 (0.63 to 63.67)
22-28 days	*	0.60 (0.14 to 2.55)		*	NA		*	5.81 (0.59 to 57.24)
Ischaemic stroke								
Baseline	10 355	1.00		6439	1.00		1069	1.00
−28 to −1 days	2671	0.67 (0.64 to 0.70)		1614	0.62 (0.58 to 0.65)		1128	4.21 (3.84 to 4.61)
0 day	43	0.29 (0.22 to 0.40)		33	0.32 (0.23 to 0.45)		273	23.55 (20.53 to 27.01)
1-7 days	968	0.94 (0.88 to 1.01)		718	0.97 (0.89 to 1.05)		326	3.94 (3.46 to 4.47)
8-14 days	1080	1.07 (1.00 to 1.14)		789	1.03 (0.95 to 1.11)		275	3.25 (2.83 to 3.72)
15-21 days	965	1.00 (0.93 to 1.07)		862	1.12 (1.04 to 1.20)		174	2.00 (1.70 to 2.35)
22-28 days	920	1.02 (0.95 to 1.10)		765	1.03 (0.96 to 1.12)		112	1.26 (1.04 to 1.53)
Myocardial infarction								
Baseline	22 079	1.00		15 124	1.00		1776	1.00
−28 to −1 days	6565	0.83 (0.81 to 0.86)		4485	0.76 (0.73 to 0.78)		1995	4.74 (4.42 to 5.08)
0 day	103	0.36 (0.29 to 0.43)		95	0.41 (0.33 to 0.50)		906	53.06 (48.77 to 57.73)
1-7 days	1881	0.92 (0.87 to 0.96)		1483	0.90 (0.85 to 0.95)		970	7.95 (7.32 to 8.63)
8-14 days	2028	0.99 (0.95 to 1.04)		1668	0.98 (0.93 to 1.03)		624	4.94 (4.50 to 5.43)
15-21 days	2000	1.02 (0.97 to 1.07)		1789	1.04 (0.99 to 1.09)		258	1.95 (1.71 to 2.22)
22-28 days	1889	1.02 (0.97 to 1.07)		1510	0.92 (0.87 to 0.97)		161	1.15 (0.97 to 1.35)
Other rare arterial thrombosis								
Baseline	1469	1.00		732	1.00		139	1.00
−28 to −1 days	365	0.63 (0.56 to 0.71)		183	0.59 (0.50 to 0.70)		164	4.70 (3.68 to 6.02)
0 day	5	0.25 (0.10 to 0.59)		*	0.08 (0.01 to 0.56)		55	35.15 (25.40 to 48.65)
1-7 days	105	0.74 (0.60 to 0.91)		93	1.03 (0.82 to 1.29)		60	5.35 (3.90 to 7.32)
8-14 days	164	1.21 (1.02 to 1.43)		88	0.95 (0.75 to 1.19)		65	5.61 (4.13 to 7.61)
15-21 days	127	1.00 (0.83 to 1.21)		107	1.16 (0.94 to 1.44)		34	2.97 (2.03 to 4.36)
22-28 days	114	1.00 (0.81 to 1.22)		102	1.15 (0.93 to 1.42)		31	2.66 (1.79 to 3.94)

BNT162b2 mRNA=Pfizer-BioNTech vaccine; ChAdOx1 nCoV-19=Oxford-AstraZeneca vaccine.

* Cells with entries <5 are suppressed.

### Primary outcomes

*Thrombocytopenia*—an increased risk was observed at 8-14 days after the ChAdOx1 nCoV-19 vaccine (incidence rate ratio 1.33, 95% confidence interval 1.19 to 1.47) and at 22-28 days (1.26, 1.13 to 1.42). An increased risk was also found after a positive SARS-CoV-2 test (1-7 days: 14.04, 12.08 to 16.31; 8-14 days: 5.27, 4.34 to 6.40; 15-21 days: 1.91, 1.44 to 2.54; 22-28 days: 1.50, 1.10 to 2.05).

*Venous thromboembolism*—an increased risk was found at 8-14 days after the ChAdOx1 nCoV-19 vaccine (incidence rate ratio 1.10, 95% confidence interval 1.02 to 1.18) and after a positive SARS-CoV-2 test (1-7 days: 13.78, 12.66 to 14.99; 8-14 days: 13.86, 12.76 to 15.05; 15-21 days: 7.88, 7.18 to 8.64; 22-28 days: 3.38, 3.00 to 3.81).

*Arterial thromboembolism*—we observed an association at 15-21 days after the BNT162b2 mRNA vaccine (incidence rate ratio 1.06, 95% confidence interval 1.01 to 1.10) and in those with SARS-CoV-2 infection (1-7 days: 6.55, 6.12 to 7.02; 8-14 days: 4.52, 4.19 to 4.88; 15-21 days: 2.02, 1.82 to 2.24; 22-28 days: 1.26, 1.11 to 1.43).

### Secondary outcomes

*CVST*—we found an increased risk at 8-14 days after the ChAdOx1 nCoV-19 vaccine (incidence rate ratio 4.01, 95% confidence interval 2.08 to 7.71), and at 15-21 days after the BNT162b2 mRNA vaccine (3.58, 1.39 to 9.27). CVST was also associated with a positive SARS-CoV-2 test (1-7 days: 12.90, 1.86 to 89.64; 8-14 days: 13.43, 1.99 to 90.59).

*Ischaemic stroke*—an increased risk was found at 15-21 days after the BNT162b2 mRNA vaccine (incidence rate ratio 1.12, 95% confidence interval 1.04 to 1.20) and after a positive SARS-CoV-2 test (1-7 days: 3.94, 3.46 to 4.47; 8-14 days: 3.25, 2.83 to 3.72; 15-21 days: 2.00, 1.70 to 2.35; 22-28 days: 1.26, 1.04 to 1.53).

*Myocardial infarction*—no association was found with the ChAdOx1 nCoV-19 or the BNT162b2 mRNA vaccine. We found an increased risk of myocardial infarction after a positive SARS-CoV-2 test (1-7 days: incidence rate ratio 7.95, 95% confidence interval 7.32 to 8.63; 8-14 days: 4.94, 4.50 to 5.43; 15-21 days: 1.95, 1.71 to 2.22).

*Other rare arterial thrombotic events*—an increased risk was observed after the ChAdOx1 nCoV-19 vaccine at 8-14 days (incidence rate ratio 1.21, 95% confidence interval 1.02 to 1.43) and after a positive SARS-CoV-2 test (1-7 days: 5.35, 3.90 to 7.32; 8-14 days: 5.61, 4.13 to 7.61; 15-21 days: 2.97, 2.03 to 4.36; 22-28 days: 2.66, 1.79 to 3.94).

### Co-occurrences of thrombocytopenia and venous thromboembolism, and of thrombocytopenia and arterial thromboembolism 

We investigated the risk of hospital admission or death from co-occurrence of thrombocytopenia and venous thromboembolism and of thrombocytopenia and arterial thromboembolism in the same people before and after the ChAdOx1 nCoV-19 or BNT162b2 mRNA vaccine within our study period. In the study period, 3606 patients were admitted to hospital for both thrombocytopenia and venous thromboembolism. Of these patients, 36 were admitted on the same day for both outcomes, while 1628 were first admitted for thrombocytopenia and subsequently for venous thromboembolism (interquartile range for the difference in admissions 16-58 days). We found an increased incidence of co-occurrence of thrombocytopenia and venous thromboembolism within 8-14 days of the ChAdOx1 nCoV-19 vaccine (incidence rate ratio 1.34, 95% confidence interval 0.99 to 1.83). No association was found with the BNT162b2 mRNA vaccine, however we observed a positive association with SARS-CoV-2 infection (1-7 days: 4.91, 3.50 to 6.89; 8-14 days: 1.91, 1.20 to 3.06; supplementary table 3).

In the study period, 2112 patients were admitted to hospital for both thrombocytopenia and arterial thromboembolism. Of these patients, 20 were admitted on the same day for both outcomes, while 1097 were first admitted for thrombocytopenia and subsequently for arterial thromboembolism (interquartile range for the difference in admissions 18-65 days). We found no association with the ChAdOx1 nCoV-19 vaccine. We found a possible signal for the BNT162b2 vaccine at 8-14 days (incidence rate ratio 1.40, 95% confidence interval 0.97 to 2.01) and a positive association with the SARS-CoV-2 infection (1-7 days: 3.69, 2.29 to 6.05; 8-14 days: 2.59, 1.47 to 4.62; supplementary table 3).

### Subgroup analyses by age group and sex

Supplementary tables 4a and 4b show the incidence rate ratios for subgroup analyses by age group or sex. An increased risk of thrombocytopenia was observed in people who had the ChAdOx1 nCoV-19 vaccine who were younger than 50 years (8-14 days: incidence rate ratio 1.56, 95% confidence interval 1.20 to 2.02; 22-28 days: 1.40, 1.04 to 1.88) and older than 50 years (8-14 days: 1.27, 1.13 to 1.43; 22-28 days: 1.22, 1.08 to 1.39). An increased risk of venous thromboembolism was observed in those who had the ChAdOx1 nCoV-19 vaccine who were older than 50 years (8-14 days: 1.09, 1.01 to 1.18) with a similar increase in those younger than 50 (1.19, 0.96 to 1.48). An increased risk of CVST was observed in those who had the ChAdOx1 nCoV-19 vaccine who were younger than 50 (8-14 days: 6.36, 2.61 to 15.46), and no significant increased risk was found in those older than 50 years. An increased risk of other rare arterial thrombosis was observed in those who had the ChAdOx1 nCoV-19 vaccine who were younger than 50 (8-14 days: 1.20, 1.01 to 1.44).

### Negative or positive control outcomes

We found no increased risk in those with coeliac disease (negative control) across the prespecified time periods for the vaccine exposures. Anaphylaxis (positive control) showed the expected increased risk in the initial period after exposure for both vaccinations but not in later periods (supplementary table 5).

### Incidence rates

The background incidence rates per 100 000 person years for thrombocytopenia and arterial thromboembolism were higher in men while rates for venous thromboembolism tended to be higher in women (supplementary tables 6a and 6b). Rates of all outcomes increased with age except for CVST where the highest rates occurred in those aged 20-50 years and 60-64 years. Rates also varied by ethnicity.

### Sensitivity analyses

Sensitivity analyses 1 and 2 were generally consistent with the main analysis. This finding suggests the results were not sensitive to censoring due to death, except for reductions in incidence rate ratios for arterial thromboembolism and ischaemic stroke associated with the BNT162b2 mRNA vaccine at 15-21 days and CVST associated with both vaccines for sensitivity analysis 2. Estimates from sensitivity analyses 3-5 were consistent with the main analysis for all outcomes, although confidence intervals were wider due to smaller numbers, particularly for CVST (supplementary tables 7a-c and supplementary fig 3a and 3b). Estimates from sensitivity analysis 6 are consistent with the main results for the ChAdOx1 nCoV-19 vaccine, while they decreased slightly for the BNT162b2 mRNA vaccine, but stayed significant for CVST.

### Absolute measures of effect of vaccinations and SARS-CoV-2 infection

We estimated the number of exposures needed for one excess event and the excess number of events per 10 million exposed to each vaccine or with a SARS-CoV-2 positive test (supplementary table 8). For example, with the ChAdOx1 nCoV-19 vaccine the excess events were 107 for thrombocytopenia, 66 for venous thromboembolism, and seven for CVST. For the BNT1262b2 mRNA vaccine, there are an estimated 143 extra cases of ischaemic stroke. For SARS-Cov-2 infection, there are an estimated 934 extra cases of thrombocytopenia, 12 614 of venous thromboembolism, 1699 of ischaemic stroke, and 20 of CVST.

## Discussion

Our analysis of serious adverse events leading to hospital admission or death, covering a population of over 29 million vaccinated people in England, showed increased relative incidences of thrombocytopenia and venous thromboembolism in the 8-14 days after ChAdOx1 nCoV-19 vaccination but not of arterial thromboembolism. Conversely, BNT162b2 mRNA vaccination was associated with arterial thromboembolism 15-21 days after vaccination but not with thrombocytopenia or venous thromboembolism. For our prespecified secondary outcomes, we found an increased risk of CVST and other rare arterial thrombotic events at 8-14 days after the ChAdOx1 nCoV-19 vaccination. We also found an increased risk of CVST and ischaemic stroke at 15-21 days after vaccination with BNT162b2 mRNA. The increased risks for CVST for both vaccines might be a potential signal, although numbers were small and further confirmation is needed. The small absolute risks associated with both of the vaccines should be noted; for instance, for 10 million people exposed to the ChAdOx1 nCoV-19 vaccine, there were seven excess events of CVST in the 28 days after the vaccine.

The subgroup analysis by age group has shown no association between CVST and the BNT162b2 mRNA vaccine, but a strong association with ChAdOx1 nCoV-19 in those younger than 50 years old (incidence rate ratio 6.36, 95% confidence interval 2.61 to 15.46). However, the sample size was considerably lower in the subgroup analyses, especially for the BNT162b2 mRNA vaccine (fewer than five people in each risk interval). However, the point estimate of incidence rate ratio associated with the BNT162b2 mRNA vaccine remained large (3.90, 0.96 to 15.82 at 15-21 days), suggesting a signal for CVST after vaccination.

The results were robust to sensitivity analyses, except for the association between the BNT162b2 mRNA vaccine and arterial thromboembolism (including ischaemic stroke), which reduced in magnitude in the sensitivity analyses accounting for fatal events. Also, we observed some variation in results for the CVST outcome; for example, the incidence rate ratios for the second sensitivity analysis were lower compared with the main analysis, which might be due to small numbers and wider confidence intervals. The incidence rate ratios associated with SARS-CoV-2 infection were much higher for each outcome than those associated with either vaccine, with the greatest risk for all outcomes being in the first week after a positive test.

### Strengths and limitations of this study

Analyses of safety signals are complex, especially in the context of a new disease for which the effects could be similar to adverse events of a vaccine deployed to prevent it. Furthermore, the vaccination programme in England began during the third pandemic wave, which coincided with the emergence of the B.1.1.7 variant of concern. In this context, case-control or cohort studies are probably subject to major confounding, especially given the pace of vaccination where matched unvaccinated controls might only be unexposed for relatively short periods before vaccination (or uninfected patients before SARS-CoV-2 infection). For this reason, we used the self-controlled case series study design, which has major advantages because the within-person comparison reduces potential confounding for all fixed characteristics. Our inclusion of calendar time in weekly blocks further accounted for temporal confounding, important given the time varying influence of pandemic waves on health and healthcare systems. To provide greater confidence in assessing associations between vaccine and outcome, we conducted our analyses on negative (coeliac) and positive (anaphylaxis) control outcomes.

The UK provides an ideal setting to study these widely deployed vaccines because they have been used at scale, enabling direct comparison. Other strengths of our study include representativeness, data completeness for exposures, and timeliness. Our results are likely to be generalisable to other older populations because we have studied a very large sample of much more diverse patients than those enrolled in clinical trials^1^ ^2^; however, they might not be generalisable to younger populations because they have not yet been vaccinated in large numbers. Unlike signals arising from case report series,^7^ ^8^ our study is not subject to recall selection biases because we used prospectively recorded medical data collected during the course of NHS clinical care. We obtained data from four, high quality, national electronic health record databases, all used for operational purposes. Our sensitivity analyses generally support our interpretations. To estimate associations with SARS-CoV-2 infection, we used people who had been vaccinated and accounted for time varying effects of vaccination and infection in the same model. We have also published our scientifically reviewed protocol.^15^

Our study is limited by restricting our analysis to first vaccine dose only (which is necessary since these analyses are being undertaken during the vaccination roll-out), lack of formal adjudication of routinely acquired outcomes, and potential for misclassification of outcomes or exposures. While we captured completed hospital admissions, we did not capture admissions where patients were still in hospital by the study end date. However, we believe that any bias, if present, is probably non-differential with respect to each vaccine, and so the comparisons between vaccines are unlikely to be affected.

### Comparison with other studies

The European Medicines Agency reported at least 169 possible cases of CVST and 53 possible cases of splanchnic vein thrombosis among 34 million recipients of the ChAdOx1 nCoV-19 vaccine; 35 possible cases of central nervous system thrombosis among 54 million recipients of the Pfizer-BioNTech mRNA vaccine; and five possible (but unvetted) cases of CVST among 4 million recipients of the Moderna mRNA vaccine. A recent study has estimated rate ratios for thromboembolic events after vaccination by using a case-control and a self-controlled case series analysis with national Scottish data.^27^ The authors found that the rate ratios estimated from the nested case-control study were larger than those estimated using the self-controlled case series analysis. This finding might indicate the presence of residual confounding or confounding by indication, which the self-controlled case series analysis takes naturally into account. Overall, their study showed broadly similar results to ours for the ChAdOx1 nCoV-19 vaccine: an increased risk of thrombocytopenia (Scottish results from self-controlled case series (0-28 days): incidence rate ratio 1.98, 95% confidence interval 1.29 to 3.02; our study (8-28 days): 1.23, 1.14 to 1.33) but not arterial thromboembolic events (0.97, 0.93 to 1.02, and 1.08, 0.95 to 1.22, respectively). The authors did not find an association between these outcomes and the BNT162b2 mRNA vaccine, as we have found. These differences might be explained by the smaller numbers in the Scottish population (0.8 million vaccinated in Scottish study with BNT162b2 mRNA compared with 9.5 million in England in our study).

### Policy implications

The increased risk of thrombocytopenia with ChAdOx1 nCoV-19 and the increased risk of arterial and venous events identified with both vaccines has major implications for healthcare policy and further research. However, these findings would benefit from corroboration from other countries using similarly robust analytical approaches and large datasets. We have highlighted the results of the statistically significant findings, although further consideration of the clinical significance of these results is needed, particularly when the estimates are close to one.

### Conclusions

We found an increased risk of thrombocytopenia, venous thromboembolism, and other rare arterial thrombotic events in short time intervals after a first dose of the ChAdOx1 nCoV-19 vaccine, and of arterial thromboembolism and ischaemic stroke after a first dose of the BNT162b2 mRNA vaccine. An increased risk of CVST was found after a first dose of both vaccines, which might be a potential signal, although numbers were small and further confirmation is needed. Importantly, the risks of these outcomes after vaccination were much lower than those associated with SARS-CoV-2 infection in the same population.

**What is already known on this topic**

Rare thrombocytopenia and thromboembolic events have occurred after covid-19 vaccination
After these rare events, several countries restricted the use of the ChAdOx1 nCoV-19 (Oxford-AstraZeneca) vaccine

**What this study adds**

Increased risk of thrombocytopenia, venous thromboembolism, and other rare arterial thrombotic events were found after a first dose of the ChAdOx1 nCoV-19 vaccine and of arterial thromboembolism and ischaemic stroke after a first dose of the BNT162b2 (Pfizer-BioNTech) vaccine
Increased risk of cerebral venous sinus thrombosis was found after a first dose of both vaccines—a week later with BNT162b2 than with ChAdOx1 nCoV-19
The risks of these outcomes after vaccination were much lower than those associated with SARS-CoV-2 infection in the same population

## Data Availability

To guarantee the confidentiality of personal and health information only the authors have had access to the data during the study in accordance with the relevant licence agreements.

## Web extra

Extra material supplied by authors

## Notes

Contributors: JHC, CACC, MP led the study conceptualisation, development of the research question, and with JD the development of advanced statistical aspects. JHC obtained data approvals, undertook the data specification, curation, and analysis, and wrote the first draft of the paper. AS, XWM, KK, SJG, DAH, PW, JD contributed to the discussion on protocol development and provided critical feedback on drafts of the manuscript. JHC, AS, PW, MP, CACC helped to edit the final manuscript. All authors approved the protocol, contributed to the critical revision of the manuscript and approved the final version of the manuscript. AZ and CACC equally contributed as senior authors. The funders of this study had no role in the design and conduct of the study and did not review or approve the manuscript. The views expressed are those of the authors and not necessarily the funders. JHC, MP, CACC had full access to all the study data, and JHC had final responsibility for submission and is the guarantor. The corresponding author attests that all listed authors meet authorship criteria and that no others meeting the criteria have been omitted.

The lead author (the manuscript’s guarantor) affirms that the manuscript is an honest, accurate, and transparent account of the study being reported; that no important aspects of the study have been omitted; and that any discrepancies from the study as planned (and, if relevant, registered) have been explained.

Dissemination to participants and related patient and public communities: This topic has gained interest from academia, policy makers, social commentators, and many parts of the media. We will disseminate using a variety of routes, such as push to traditional press and on social media platforms. A lay summary will be made available on the QResearch websites and newsletter.

Provenance and peer review: Not commissioned; externally peer reviewed.
