# Supplementary material for: Risk of thrombocytopenia and thromboembolism after covid-19 vaccination and SARS-CoV-2 positive testing: self-controlled case series study
Source: BMJ. 2021 Aug 26;374:n1931. doi: 10.1136/bmj.n1931 (PMC8388189; doi:10.1136/bmj.n1931)
Supplement: Supplementary file 1 — Supplementary information: additional figures 1 to 3b and tables 1 to 8 [file hipj066873.ww.pdf]

Supplementary figure 1: Self-controlled case series study design. Each patient is followed from index date to study end date and censored if dead or had second dose of vaccine. Only patients who had experienced the outcome are observed. Each patient can experience the outcome in one of the following intervals: the baseline interval (29 days or more before exposure date), pre-risk interval (1 to 28 days before exposure date) or one of the risk interval post-exposure. We considered three exposures: Oxford/AstraZeneca vaccine, Pfizer/BioNTech vaccine, positive SARS-CoV-2 test.

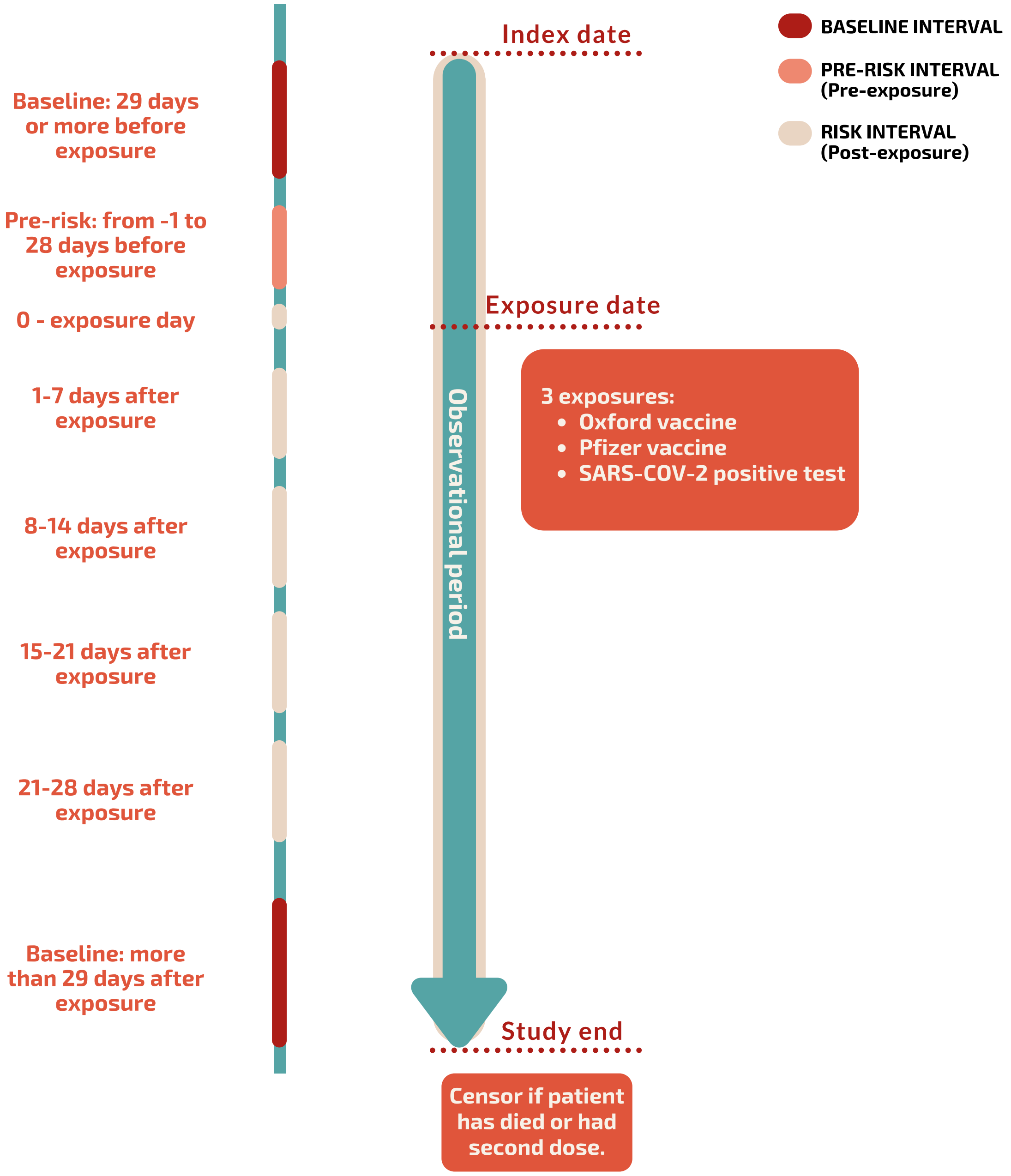

Supplementary Figure 2a

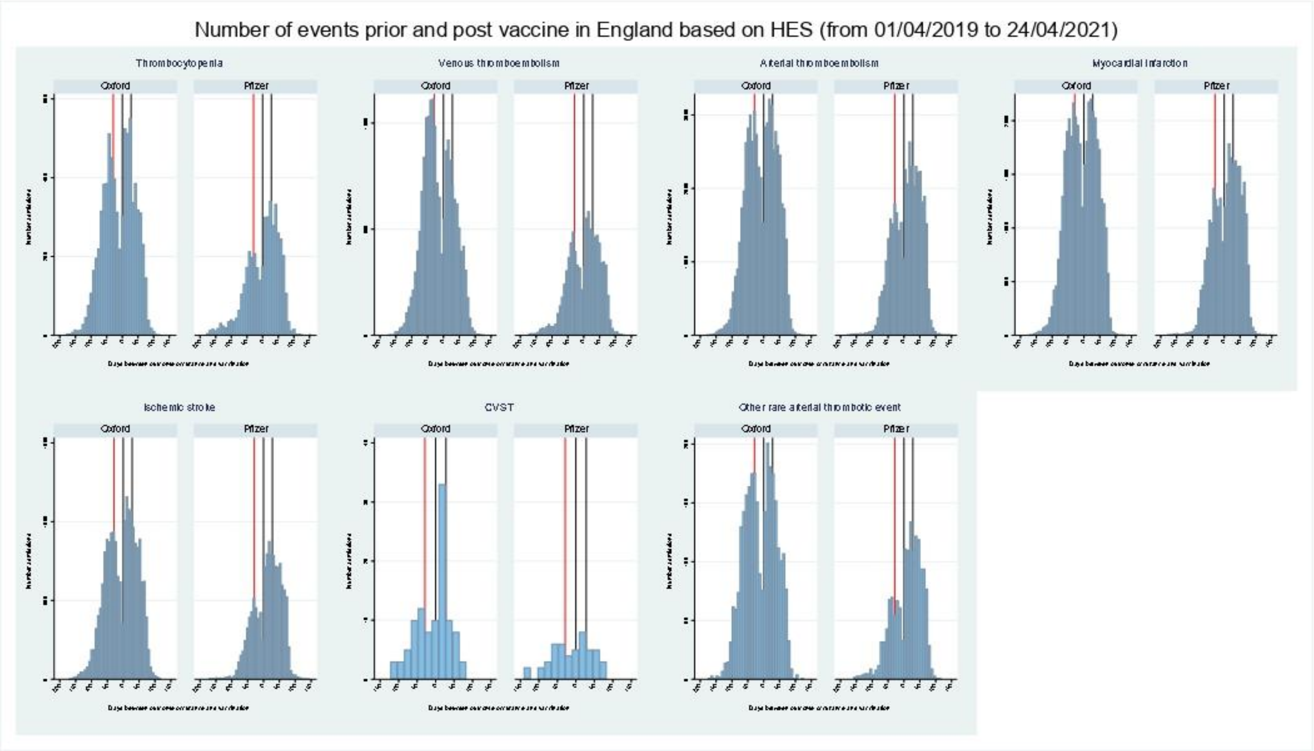

Supplementary Figure 2b

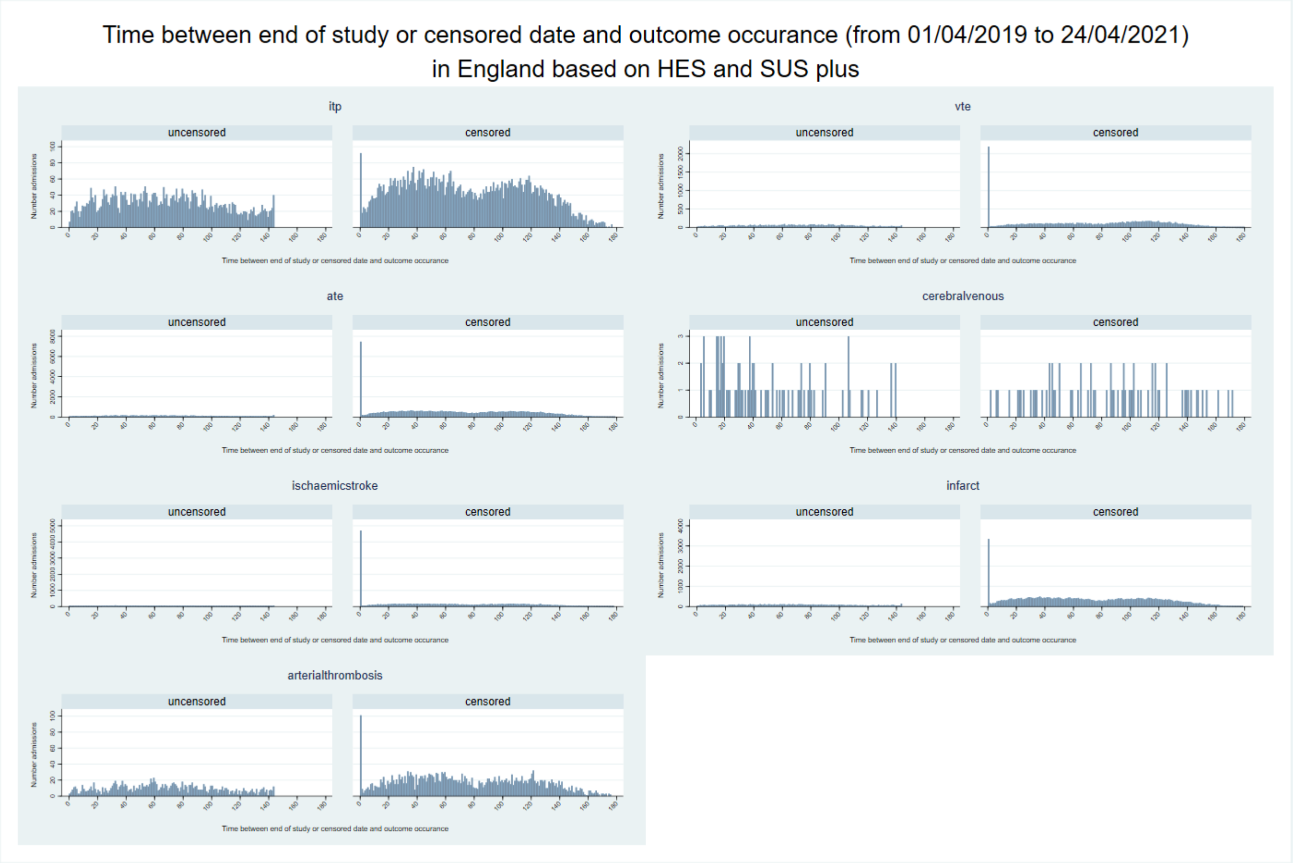

Supplementary figure 3a: Incidence rate ratios (IRR 95% CI) for outcomes in pre-defined risk periods immediately before and after exposure to the ChAdOx1nCoV-19 vaccination and before and after a positive SARS-CoV-2 test result from December 1, 2020 to April 24, 2021. Comparisons between different sensitivity analyses.

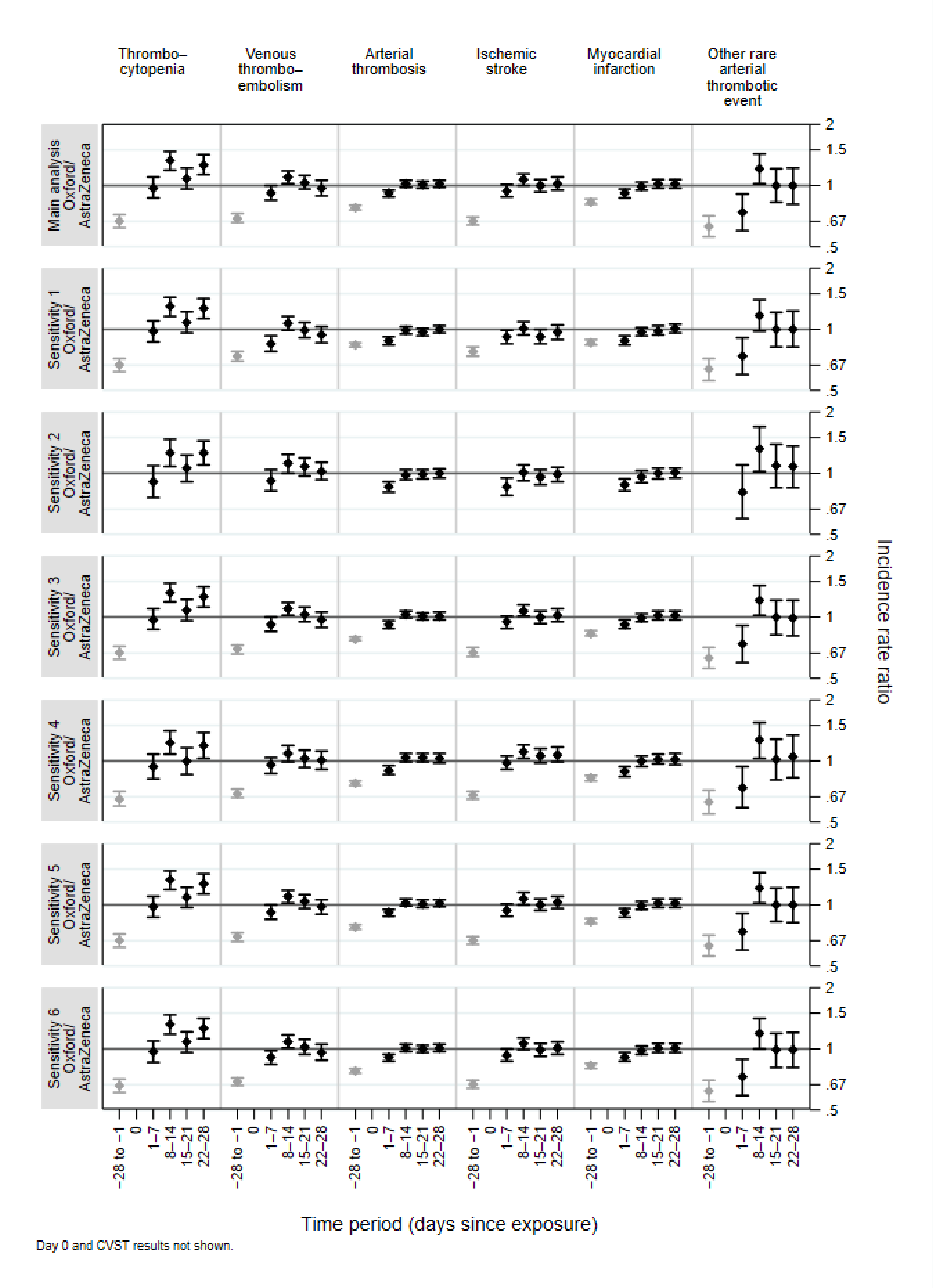

Supplementary figure 3b: Incidence rate ratios (IRR 95% CI) for outcomes in pre-defined risk periods immediately before and after exposure to the BNT162b2 mRNA vaccination and before and after a positive SARS-CoV-2 test result from December 1, 2020 to April 24, 2021. Comparisons between different sensitivity analyses.

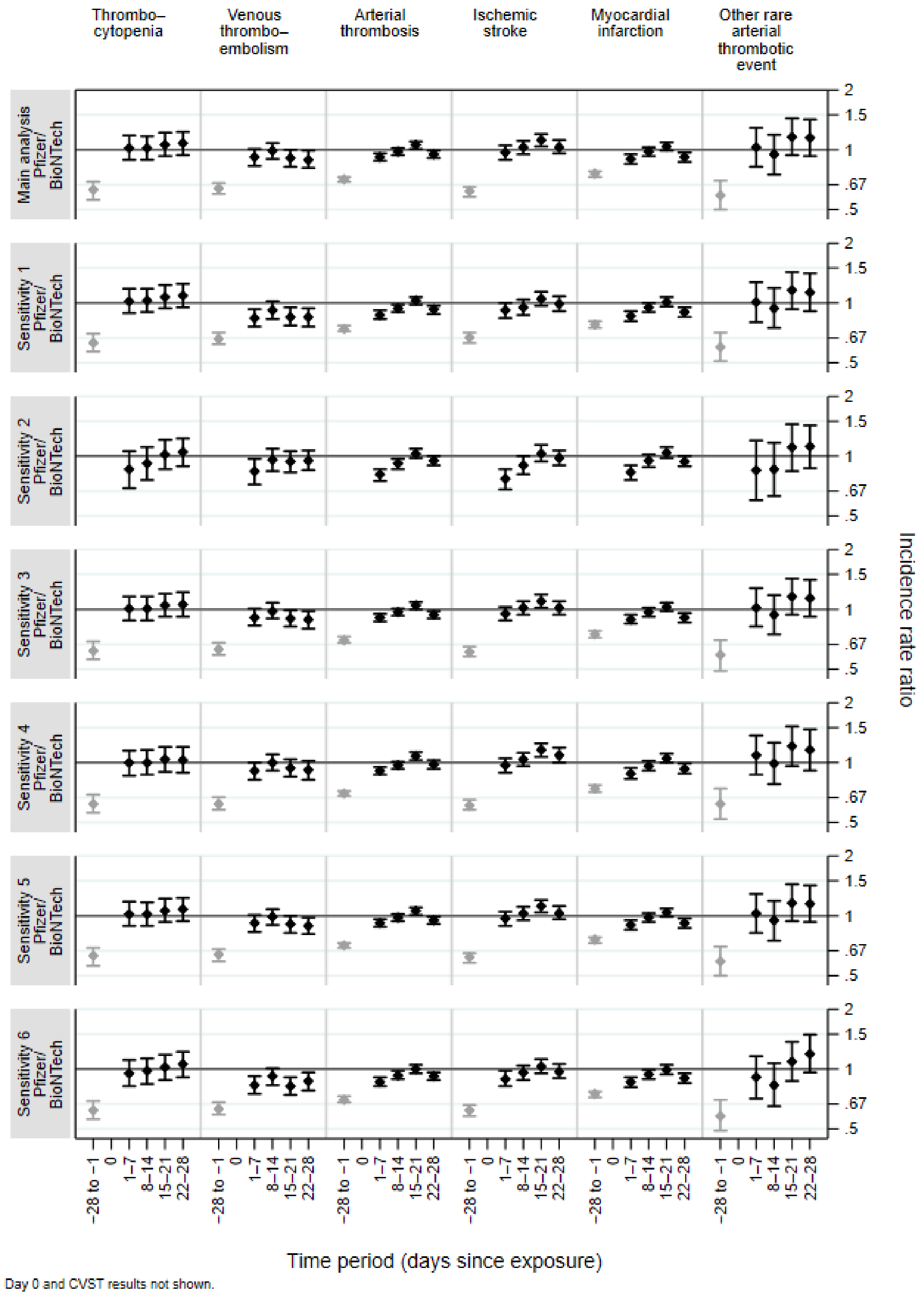

Supplementary table 1: ICD-10 codes used to identify cases with each outcome.

Outcome	ICD-10 code and description
**Thrombocytopenia**	D693 - Idiopathic thrombocytopenic purpura
	D694 - Other primary thrombocytopenia
	D695 - Secondary thrombocytopenia
	D696 - Thrombocytopenia, unspecified
**Venous thromboembolism**	I26 - Pulmonary embolism
	I260 - Pulmonary embolism with mention of acute cor pulmonale
	I269 - Pulmonary embolism without mention of acute cor pulmonale
	I81 - Portal vein thrombosis
	I81X - Portal vein thrombosis
	I82 - Other venous embolism and thrombosis
	I820 - Budd-Chiari syndrome
	I822 - Embolism and thrombosis of vena cava
	I823 - Embolism and thrombosis of renal vein
	I828 - Embolism and thrombosis of other specified veins
	I829 - Embolism and thrombosis of unspecified vein
**Arterial thrombosis**	I74 - Arterial embolism and thrombosis
	I740 - Embolism and thrombosis of abdominal aorta
	I741 - Embolism and thrombosis of other and unspecified parts of aorta
	I742 - Embolism and thrombosis of arteries of upper extremities
	I743 - Embolism and thrombosis of arteries of lower extremities
	I744 - Embolism and thrombosis of arteries of extremities, unspecified
	I745 - Embolism and thrombosis of iliac artery
	I748 - Embolism and thrombosis of other arteries

	I749 - Embolism and thrombosis of unspecified artery
**Cerebral venous sinus thrombosis**	O225 - Cerebral venous thrombosis in pregnancy
	O873 - Cerebral venous thrombosis in the puerperium
	I636 - Cerebral infarction due to cerebral venous thrombosis
	I676 – Non-pyogenic thrombosis of intracranial venous system
**Ischaemic stroke**	G45 - Transient cerebral ischaemic attacks and related syndromes
	G450 - Vertebrobasilar artery syndrome
	G451 - Carotid artery syndrome (hemispheric)
	G452 - Multiple and bilateral precerebral artery syndromes
	G453 - Amaurosis fugax
	G454 - Transient global amnesia
	G458 - Other transient cerebral ischaemic attacks and related syndromes
	G459 - Transient cerebral ischaemic attack, unspecified
	I63 - Cerebral infarction
	I630 - Cerebral infarction due to thrombosis of precerebral arteries
	I631 - Cerebral infarction due to embolism of precerebral arteries
	I632 - Cerebral infarction due to unspecified occlusion or stenosis of precerebral arteries
	I633 - Cerebral infarction due to thrombosis of cerebral arteries
	I634 - Cerebral infarction due to embolism of cerebral arteries
	I635 - Cerebral infarction due to unspecified occlusion or stenosis of cerebral arteries
	I636 - Cerebral infarction due to cerebral venous thrombosis, non-pyogenic
	I638 - Other cerebral infarction
	I639 - Cerebral infarction, unspecified
	I64 - Stroke, not specified as haemorrhage or infarction
	I64X - Stroke, not specified as haemorrhage or infarction
	I21 - Acute myocardial infarction

**Myocardial infarction**	I210 - Acute transmural myocardial infarction of anterior wall
	I211 - Acute transmural myocardial infarction of inferior wall
	I212 - Acute transmural myocardial infarction of other sites
	I213 - Acute transmural myocardial infarction of unspecified site
	I214 - Acute subendocardial myocardial infarction
	I219 - Acute myocardial infarction, unspecified
	I22 - Subsequent myocardial infarction
	I220 - Subsequent myocardial infarction of anterior wall
	I221 - Subsequent myocardial infarction of inferior wall
	I228 - Subsequent myocardial infarction of other sites
	I229 - Subsequent myocardial infarction of unspecified site
	I23 - Certain current complications following acute myocardial infarction
	I230 – Haemo-pericardium as current complication following acute myocardial infarction
	I231 - Atrial septal defect as current complication following acute myocardial infarction
	I232 - Ventricular septal defect as current complication following acute myocardial infarction
	I233 - Rupture of cardiac wall without haemopericardium as current complication following acute myocardial
	I234 - Rupture of chordae tendineae as current complication following acute myocardial infarction
	I235 - Rupture of papillary muscle as current complication following acute myocardial infarction
	I236 - Thrombosis of atrium, auricular appendage, and ventricle as current complications following acute myocardial
	I238 - Other current complications following acute myocardial infarction
	I252 - Old myocardial infarction

Supplementary table 2: Demographic characteristics of patients who experienced one of the secondary outcomes in the 28 days following a COVID-19 vaccine first dose or SARS-COV-2 positive test in England amongst the vaccinated population from December 1, 2020 until April 24, 2021 (cells with < 5 are suppressed).

	Cerebral venous sinus thrombosis (CVST)			Ischaemic Stroke			Myocardial infarction			Other Arterial Thrombosis		
	ChAdOx1 nCoV-19 vaccine	BNT162b2 mRNA vaccine	Positive SARS-CoV-2 test	ChAdOx1nCoV-19 vaccine	BNT162b2 mRNA vaccine	Positive SARS-CoV-2 test	ChAdOx1nCoV-19 vaccine	BNT162b2 mRNA vaccine	Positive SARS-CoV-2 test	ChAdOx1 nCoV-19 vaccine	BNT162b2 mRNA vaccine	Positive SARS-CoV-2 test
Total number of people	28	10	8	3976	3167	1160	7901	6544	2919	515	391	245
Women	60.7 (17)	70.0 (7)	62.5 (5)	50.2 (1997)	50.1 (1586)	44.4 (515)	33.6 (2654)	33.8 (2211)	31.1 (908)	41.2 (212)	37.3 (146)	35.1 (86)
Men	39.3 (11)	30.0 (3)	37.5 (3)	49.8 (1979)	49.9 (1579)	55.6 (645)	66.3 (5237)	66.2 (4329)	68.9 (2011)	58.8 (303)	62.7 (245)	64.9 (159)
Not recorded	0	0	0	0	0.1 (2)	0	0.1 (10)	0.1 (4)	0	0	0	0
Mean age (SD)	49.6 (20.3)	60.5 (20.6)	66.5 (9.6)	74.8 (12.9)	78.8 (11.3)	75.0 (14.2)	71.3 (12.1)	75.9 (11.7)	71.8 (13.1)	71.4 (12.2)	75.4 (11.2)	69.2 (13.2)
16-29 years	21.4 (6)	0	0	0.2 (7)	*	*	0.1 (7)	*	0	0	0	*
30-39 years	17.9 (5)	*	0	0.6 (25)	0.4 (14)	<10	0.5 (37)	<20	0.5 (14)	1.0 (5)	0	*
40-49 years	*	*	0	2.2 (86)	1.3 (41)	4.7 (55)	2.8 (222)	1.7 (111)	4.1 (120)	3.1 (16)	2.3 (9)	4.5 (11)
50-59 years	25.0 (7)	*	*	9.8 (388)	4.8 (152)	10.9 (127)	14.1 (1114)	8.8 (574)	14.7 (429)	12.8 (66)	7.4 (29)	18.0 (44)
60-69 years	*	*	*	18.4 (732)	11.4 (360)	14.8 (172)	24.9 (1965)	16.6 (1088)	23.8 (694)	24.1 (124)	18.9 (74)	23.3 (57)

70-79 years	*	*	*	32.0 (1272)	24.8 (784)	23.4 (271)	34.1 (2696)	26.9 (1761)	25.4 (740)	34.6 (178)	30.7 (120)	29.8 (73)
80-89 years	*	*	0	23.4 (931)	42.7 (1351)	29.9 (347)	16.1 (1271)	36.5 (2386)	23.1 (675)	17.5 (90)	34.0 (133)	18.8 (46)
90+ years	*	0	0	13.5 (535)	14.6 (461)	15.3 (177)	7.5 (589)	9.2 (605)	8.5 (247)	7.0 (36)	6.6 (26)	4.1 (10)
White	82.1 (23)	100.0 (10)	62.5 (5)	88.7 (3526)	89.7 (2841)	82.8 (961)	88.6 (7004)	89.6 (5866)	79.7 (2325)	90.3 (465)	92.6 (362)	85.3 (209)
Indian	0	0	0.0 (0)	2.1 (85)	1.8 (57)	2.8 (33)	2.6 (204)	2.8 (186)	4.9 (143)	1.4 (7)	1.5 (6)	1.6 (4)
Pakistani	0	0	0.0 (0)	1.2 (46)	0.8 (26)	3.2 (37)	1.9 (153)	1.5 (98)	4.5 (132)	*	0	2.0 (5)
Bangladeshi	*	0	*	0.3 (10)	<10	1.1 (13)	0.5 (38)	0.4 (23)	1.6 (47)	*	0	*
Other Asian	*	0	0.0 (0)	0.6 (24)	0.4 (13)	<10	0.6 (51)	0.7 (44)	2.0 (59)	*	0	*
Black Caribbean	0	0	0.0 (0)	1.0 (39)	0.8 (26)	1.3 (15)	0.6 (44)	0.3 (21)	0.9 (26)	1.0 (5)	1.3 (5)	*
Black African	*	0	0.0 (0)	0.7 (28)	0.5 (16)	1.8 (21)	0.4 (35)	0.4 (26)	0.7 (20)	*	*	*
Chinese	0	0	0.0 (0)	0.2 (6)	*	*	0.1 (11)	0.1 (8)	0.3 (10)	0	*	0
Other ethnic group	0	0	0.0 (0)	1.0 (39)	0.9 (27)	2.1 (24)	1.1 (89)	1.0 (66)	2.5 (72)	*	0	3.7 (9)
Not recorded	7.1 (2)	0	*	4.4 (173)	4.7 (149)	3.8 (44)	3.4 (272)	3.1 (206)	2.9 (85)	4.7 (24)	3.6 (14)	3.3 (8)

Supplementary table 3: Incidence rate ratios (IRR 95% CI) for the two composite outcomes: thrombocytopenia and venous thromboembolism; thrombocytopenia and arterial thromboembolism in pre-defined risk periods immediately before and after exposure to vaccination and before and after a positive SARS-CoV-2 test result, adjusted for calendar time from December 1, 2020 to April 24, 2021

		ChAdOx1nCoV-19 vaccine		BNT162b2 mRNA vaccine		Positive SARS-CoV-2 test	
	**Time period**	**events**	**IRR (99% CI)**	**events**	**IRR (99% CI)**	**events**	**IRR (99% CI)**
**Composite primary outcomes**							
**Thrombocytopenia + Venous thromboembolism**	baseline	1761	1.00	1755	1.00	1927	1.00
	-28 to -1 days	261	0.87 (0.74, 1.03)	234	0.93 (0.78, 1.10)	123	2.45 (1.83, 3.27)
	0 day	*	0.51 (0.19, 1.37)	10	1.22 (0.65, 2.31	39	21.40 (14.47, 31.66)
	1-7 days	63	1.27 (0.95, 1.68)	55	0.99 (0.73, 1.34)	63	4.91 (3.50, 6.89)
	8-14 days	53	1.34 (0.99, 1.83)	56	1.11 (0.82, 1.50)	23	1.91 (1.20, 3.06)
	15-21 days	25	0.83 (0.54, 1.27)	48	1.13 (0.82, 1.56)	12	1.09 (0.60, 2.01)
	22-28 days	30	1.38 (0.93, 2.06	39	1.24 (0.87, 1.76)	10	0.98 (0.51, 1.90)
**Thrombocytopenia + Arterial thromboembolism**	baseline	1057	1.00	1054	1.00	1136	1.00
	-28 to -1 days	133	0.83 (0.66, 1.02)	122	0.89 (0.71, 1.13)	88	3.32 (2.31, 4.76)
	0 day	*	0.89 (0.34, 2.40)	*	0.46 (0.11, 1.84)	20	20.55 (12.07, 33.05)
	1-7 days	29	0.97 (0.65, 1.46)	30	1.00 (0.67, 1.49)	25	3.69 (2.29, 6.05)
	8-14 days	32	1.28 (0.87, 1.90)	39	1.40 (0.97, 2.01)	16	2.59 (1.47, 4.62)
	15-21 days	21	1.02 (0.64, 1.90)	30	1.27 (0.85, 1.91)	8	1.38 (0.65, 2.93)
	22-28 days	23	1.43 (0.91, 2.25	22	1.14 (0.72, 1.81)	6	1.13 (0.48, 2.64)

Supplementary table 4a: Incidence rate ratios (IRR 95% CI) by age groups for primary composite and secondary outcomes in pre-defined risk periods immediately before and after exposure to vaccination and before and after a positive SARS-CoV-2 test result, adjusted for calendar time from December 1, 2020 to April 24, 2021

		ChAdOx1nCoV-19 vaccine		BNT162b2 mRNA vaccine		Positive SARS-CoV-2 test	
	Time period	events	IRR (95% CI)	events	IRR (95% CI)	events	IRR (95% CI)
Composite primary outcomes							
Thrombocytopenia							
Age ≤ 50	baseline	759	1.00	256	1.00	73	1.00
	-28 to -1 days	194	0.85 (0.71, 1.01)	82	0.91 (0.70, 1.17)	50	2.82 (1.92, 4.12)
	0 day	*	0.40 (0.13, 1.25)	*	1.23 (0.46, 3.34)	28	39.05 (24.87, 61.31)
	1-7 days	48	0.96 (0.71, 1.30)	20	0.89 (0.56, 1.43)	61	11.78 (8.23, 16.84)
	8-14 days	74	1.56 (1.20, 2.02)	21	0.98 (0.62, 1.55)	24	4.53 (2.82, 7.27)
	15-21 days	56	1.28 (0.96, 1.71)	27	1.31 (0.87, 1.98)	13	2.34 (1.29, 4.25)
	22-28 days	54	1.40 (1.04, 1.88)	27	1.33 (0.88, 2.01)	7	1.25 (0.57, 2.72)
Age > 50	baseline	3092	1.00	1753	1.00	308	1.00
	-28 to -1 days	716	0.63 (0.57, 0.68)	422	0.59 (0.52, 0.66)	380	5.15 (4.37, 6.06)
	0 day	16	0.39 (0.24, 0.64)	9	0.31 (0.16, 0.60)	271	83.58 (70.17, 99.56)
	1-7 days	283	0.97 (0.85, 1.10)	223	1.02 (0.88, 1.19)	337	14.47 (12.26, 17.08)
	8-14 days	364	1.27 (1.13, 1.43)	233	1.01 (0.88, 1.17)	128	5.41 (4.37, 6.69)
	15-21 days	281	1.04 (0.91, 1.18)	232	1.03 (0.89, 1.19)	43	1.80 (1.30, 2.49)
	22-28 days	302	1.22 (1.08, 1.39)	214	1.04 (0.89, 1.20)	38	1.55 (1.10, 2.17)
Venous thromboembolism							
Age ≤ 50	baseline	1701	1.00	585	1.00	162	1.00
	-28 to -1 days	416	0.83 (0.73, 0.94)	162	0.67 (0.55, 0.80)	98	2.34 (1.79, 3.06)
	0 day	6	0.43 (0.19, 0.95)	*	0.42 (0.14, 1.31)	87	47.64 (36.25, 62.61)
	1-7 days	67	0.71 (0.55, 0.92)	40	0.79 (0.57, 1.10)	202	15.44 (12.38, 19.27)
	8-14 days	102	1.19 (0.96, 1.48)	40	0.79 (0.57, 1.11)	290	21.24 (17.30, 26.08)

	15-21 days	94	1.25 (0.99, 1.57)	28	0.56 (0.38, 0.83)	193	13.53 (10.88, 16.83)
	22-28 days	56	0.89 (0.67, 1.18)	24	0.55 (0.36, 0.83)	48	3.30 (2.38, 4.58)
**Age > 50**	baseline	8145	1.00	4042	1.00	1049	1.00
	-28 to -1 days	2145	0.68 (0.65, 0.72)	1062	0.64 (0.59, 0.68)	909	3.61 (3.28, 3.98)
	0 day	47	0.47 (0.35, 0.62)	22	0.34 (0.23, 0.52)	746	65.97 (59.65, 72.96)
	1-7 days	679	0.95 (0.87, 1.03)	446	0.94 (0.84, 1.04)	1103	13.49 (12.31, 14.79)
	8-14 days	768	1.09 (1.01, 1.18)	515	1.01 (0.92, 1.12)	1081	12.68 (11.58, 13.89)
	15-21 days	669	1.01 (0.93, 1.10)	486	0.94 (0.86, 1.04)	614	6.97 (6.28, 7.73)
	22-28 days	589	0.98 (0.90, 1.07)	450	0.93 (0.84, 1.03)	307	3.39 (2.98, 3.86)
**Arterial thromboembolism**							
**Age ≤ 50**	baseline	2316	1.00	800	1.00	190	1.00
	-28 to -1 days	703	0.93 (0.84, 1.03)	251	0.79 (0.68, 0.91)	112	2.29 (1.79, 2.94)
	0 day	5	0.21 (0.09, 0.50)	*	0.17 (0.04, 0.70)	62	32.05 (23.80, 43.16)
	1-7 days	110	0.67 (0.55, 0.82)	45	0.57 (0.42, 0.77)	81	5.74 (4.38, 7.52)
	8-14 days	155	1.02 (0.86, 1.22)	61	0.79 (0.60, 1.03)	65	4.42 (3.31, 5.91)
	15-21 days	133	1.00 (0.83, 1.21)	65	0.86 (0.66, 1.12)	33	2.22 (1.52, 3.23)
	22-28 days	103	0.91 (0.74, 1.12)	69	0.97 (0.75, 1.25)	14	0.90 (0.52, 1.55)
**Age > 50**	baseline	29628	1.00	20347	1.00	2553	1.00
	-28 to -1 days	8452	0.77 (0.75, 0.80)	5733	0.71 (0.69, 0.73)	2942	4.77 (4.50, 5.05)
	0 day	138	0.34 (0.29, 0.41)	123	0.39 (0.32, 0.46)	1103	43.01 (39.95, 46.31)
	1-7 days	2659	0.93 (0.90, 0.97)	2116	0.93 (0.89, 0.98)	1209	6.61 (6.16, 7.10)
	8-14 days	2910	1.03 (0.99, 1.07)	2316	0.99 (0.94, 1.03)	852	4.53 (4.18, 4.90)
	15-21 days	2773	1.02 (0.98, 1.06)	2525	1.07 (1.02, 1.11)	393	2.01 (1.80, 2.24)
	22-28 days	2631	1.02 (0.98, 1.07)	2152	0.95 (0.91, 1.00)	264	1.29 (1.13, 1.46)
**Secondary outcomes**							
**Cerebral venous sinus thrombosis (CVST)**							
**Age ≤ 50**	baseline	23	1.00	9	1.00	*	1.00

	-28 to -1 days	*	0.56 (0.18, 1.74)	*	0.87 (0.23, 3.30)	*	n/a
	0 day	*	n/a	*	n/a	*	n/a
	1-7 days	*	n/a	*	n/a	*	n/a
	8-14 days	11	6.36 (2.61, 15.46)	*	1.40 (0.17, 11.79)	*	n/a
	15-21 days	*	1.92 (0.51, 7.19)	*	3.90 (0.96, 15.82)	*	n/a
	22-28 days	*	0.73 (0.09, 5.85)	*	n/a	*	n/a
**Age > 50**	baseline	21	1.00	12	1.00	*	1.00
	-28 to -1 days	5	0.80 (0.27, 2.36)	*	1.22 (0.37, 4.09)	*	n/a
	0 day	*	4.63 (0.58, 36.74)	*	n/a	*	n/a
	1-7 days	*	1.19 (0.25, 5.53)	*	n/a	*	n/a
	8-14 days	*	2.54 (0.78, 8.25)	*	3.43 (0.89, 13.18)	*	n/a
	15-21 days	5	2.74 (0.92, 8.18)	*	3.23 (0.85, 12.27)	*	n/a
	22-28 days	*	0.58 (0.07, 4.53)	*	n/a	*	n/a
**Ischaemic stroke**							
**Age ≤ 50**	baseline	692	1.00	223	1.00	57	1.00
	-28 to -1 days	205	0.90 (0.75, 1.08)	53	0.59 (0.44, 0.81)	41	2.80 (1.82, 4.32)
	0 day	1	0.13 (0.02, 0.96)	2	0.67 (0.16, 2.70)	23	38.34 (23.10, 63.63)
	1-7 days	26	0.52 (0.34, 0.78)	14	0.63 (0.36, 1.10)	20	4.55 (2.69, 7.71)
	8-14 days	45	0.99 (0.72, 1.37)	16	0.72 (0.43, 1.21)	17	3.77 (2.16, 6.58)
	15-21 days	39	0.99 (0.70, 1.40)	18	0.80 (0.49, 1.32)	12	2.60 (1.38, 4.91)
	22-28 days	35	1.06 (0.74, 1.53)	19	0.92 (0.57, 1.50)	7	1.46 (0.66, 3.21)
**Age > 50**	baseline	9663	1.00	6216	1.00	1012	1.00
	-28 to -1 days	2466	0.66 (0.63, 0.69)	1561	0.62 (0.58, 0.66)	1087	4.28 (3.90, 4.70)
	0 day	42	0.30 (0.22, 0.41)	31	0.31 (0.22, 0.44)	250	22.69 (19.67, 26.18)
	1-7 days	942	0.96 (0.90, 1.03)	704	0.98 (0.90, 1.06)	306	3.89 (3.41, 4.44)
	8-14 days	1035	1.08 (1.01, 1.15)	773	1.04 (0.96, 1.12)	258	3.21 (2.79, 3.70)
	15-21 days	926	1.00 (0.93, 1.08)	844	1.13 (1.05, 1.22)	162	1.97 (1.66, 2.33)
	22-28 days	885	1.02 (0.95, 1.10)	746	1.04 (0.96, 1.12)	105	1.25 (1.02, 1.53)

**Myocardial infarction**							
**Age ≤ 50**	baseline	1555	1.00	561	1.00	125	1.00
	-28 to -1 days	486	0.96 (0.85, 1.08)	196	0.88 (0.74, 1.04)	76	2.41 (1.78, 3.27)
	0 day	*	0.25 (0.09, 0.66)	0	n/a	37	29.99 (20.50, 43.86)
	1-7 days	84	0.77 (0.61, 0.97)	31	0.56 (0.39, 0.81)	56	6.24 (4.49, 8.67)
	8-14 days	106	1.04 (0.84, 1.28)	45	0.84 (0.62, 1.16)	45	4.77 (3.36, 6.78)
	15-21 days	88	0.97 (0.77, 1.22)	45	0.88 (0.64, 1.20)	21	2.19 (1.37, 3.50)
	22-28 days	66	0.85 (0.66, 1.11)	49	1.01 (0.75, 1.37)	7	0.69 (0.32, 1.49)
**Age > 50**	baseline	20524	1.00	14563	1.00	1651	1.00
	-28 to -1 days	6079	0.83 (0.80, 0.85)	4289	0.75 (0.73, 0.78)	1919	4.92 (4.58, 5.29)
	0 day	99	0.37 (0.30, 0.45)	95	0.43 (0.35, 0.52)	869	54.86 (50.31, 59.83)
	1-7 days	1797	0.93 (0.89, 0.98)	1452	0.92 (0.87, 0.97)	914	8.09 (7.43, 8.81)
	8-14 days	1922	1.00 (0.95, 1.05)	1623	0.99 (0.94, 1.04)	579	4.96 (4.49, 5.46)
	15-21 days	1912	1.02 (0.98, 1.08)	1744	1.05 (1.00, 1.10)	237	1.93 (1.68, 2.22)
	22-28 days	1823	1.03 (0.98, 1.08)	1461	0.92 (0.87, 0.98)	154	1.18 (1.00, 1.40)
**Other rare arterial thrombosis**							
**Age ≤ 50**	baseline	125	1.00	41	1.00	12	1.00
	-28 to -1 days	30	0.80 (0.51, 1.27)	8	0.47 (0.21, 1.04)	*	0.46 (0.10, 2.10)
	0 day	*	n/a	*	n/a	*	19.21 (5.23, 70.50)
	1-7 days	6	0.79 (0.33, 1.89)	*	0.28 (0.04, 2.04)	8	7.09 (2.79, 17.99)
	8-14 days	8	1.19 (0.54, 2.60)	*	0.52 (0.12, 2.22)	5	4.29 (1.47, 12.55)
	15-21 days	7	1.24 (0.54, 2.85)	*	0.98 (0.34, 2.83)	*	n/a
	22-28 days	5	1.02 (0.39, 2.68)	*	0.51 (0.12, 2.17)	*	0.95 (0.12, 7.34)
**Age > 50**	baseline	1344	1.00	691	1.00	127	1.00
	-28 to -1 days	335	0.62 (0.54, 0.70)	175	0.60 (0.50, 0.72)	162	5.27 (4.08, 6.79)
	0 day	5	0.26 (0.11, 0.62)	*	0.08 (0.01, 0.59)	52	37.12 (26.52, 51.97)
	1-7 days	99	0.73 (0.59, 0.91)	92	1.07 (0.85, 1.34)	52	5.18 (3.70, 7.25)

	8-14 days	156	1.20 (1.01, 1.44)	86	0.96 (0.76, 1.21)	60	5.77 (4.19, 7.93)
	15-21 days	120	0.98 (0.81, 1.20)	103	1.17 (0.94, 1.45)	34	3.30 (2.24, 4.86)
	22-28 days	109	0.99 (0.80, 1.21)	100	1.17 (0.95, 1.46)	30	2.84 (1.90, 4.25)

Supplementary table 4b: Incidence rate ratios (IRR 95% CI) by sex for primary composite and secondary outcomes in pre-defined risk periods immediately before and after exposure to vaccination and before and after a positive SARS-CoV-2 test result, adjusted for calendar time from December 1, 2020 to April 24, 2021.

		ChAdOx1nCoV-19 vaccine		BNT162b2 mRNA vaccine		Positive SARS-CoV-2 test	
	**Time period**	**events**	**IRR (95% CI)**	**events**	**IRR (95% CI)**	**events**	**IRR (95% CI)**
**Composite primary outcomes**							
**Thrombocytopenia**							
**Female**	baseline	1832	1.00	899	1.00	190	1.00
	-28 to -1 days	420	0.64 (0.57, 0.72)	246	0.69 (0.59, 0.80)	198	4.37 (3.52, 5.43)
	0 day	7	0.30 (0.14, 0.64)	5	0.37 (0.15, 0.89)	117	60.51 (47.37, 77.28)
	1-7 days	156	0.97 (0.81, 1.15)	109	1.09 (0.88, 1.34)	154	11.22 (8.95, 14.07)
	8-14 days	221	1.40 (1.21, 1.63)	103	0.98 (0.79, 1.21)	61	4.41 (3.28, 5.94)
	15-21 days	159	1.07 (0.91, 1.28)	116	1.12 (0.91, 1.36)	24	1.72 (1.12, 2.64)
	22-28 days	165	1.22 (1.03, 1.44)	106	1.09 (0.89, 1.35)	19	1.32 (0.82, 2.12)
**Male**	baseline	2019	1.00	1110	1.00	191	1.00
	-28 to -1 days	490	0.69 (0.62, 0.76)	258	0.58 (0.50, 0.67)	232	5.06 (4.12, 6.23)
	0 day	12	0.47 (0.27, 0.84)	8	0.43 (0.21, 0.87)	182	90.64 (73.01, 112.54)
	1-7 days	175	0.98 (0.83, 1.16)	134	0.97 (0.80, 1.18)	244	16.75 (13.68, 20.51)
	8-14 days	217	1.26 (1.08, 1.46)	151	1.05 (0.88, 1.26)	91	6.08 (4.69, 7.86)
	15-21 days	178	1.09 (0.93, 1.29)	143	1.02 (0.85, 1.23)	32	2.09 (1.43, 3.05)
	22-28 days	191	1.30 (1.11, 1.53)	135	1.06 (0.88, 1.28)	26	1.68 (1.11, 2.53)

**Venous thromboembolism**							
**Female**	baseline	4562	1.00	2299	1.00	620	1.00
	-28 to -1 days	1176	0.67 (0.62, 0.72)	576	0.61 (0.55, 0.67)	491	3.33 (2.93, 3.79)
	0 day	27	0.47 (0.32, 0.68)	12	0.33 (0.19, 0.58)	333	50.55 (43.87, 58.24)
	1-7 days	380	0.93 (0.83, 1.04)	244	0.91 (0.80, 1.05)	508	10.69 (9.43, 12.13)
	8-14 days	466	1.17 (1.05, 1.29)	278	0.98 (0.86, 1.12)	495	10.02 (8.84, 11.36)
	15-21 days	380	1.02 (0.91, 1.14)	271	0.96 (0.84, 1.09)	297	5.83 (5.05, 6.73)
	22-28 days	352	1.04 (0.93, 1.17)	236	0.88 (0.77, 1.01)	162	3.10 (2.60, 3.70)
**Male**	baseline	5279	1.00	2324	1.00	591	1.00
	-28 to -1 days	1385	0.72 (0.68, 0.77)	648	0.66 (0.60, 0.73)	516	3.54 (3.11, 4.02)
	0 day	26	0.45 (0.31, 0.67)	13	0.37 (0.22, 0.64)	500	76.51 (67.35, 86.92)
	1-7 days	362	0.90 (0.81, 1.01)	241	0.92 (0.80, 1.06)	797	16.85 (15.02, 18.90)
	8-14 days	403	1.04 (0.93, 1.16)	277	1.00 (0.88, 1.14)	876	17.67 (15.80, 19.76)
	15-21 days	382	1.06 (0.95, 1.18)	243	0.86 (0.75, 0.99)	510	9.89 (8.74, 11.19)
	22-28 days	289	0.90 (0.79, 1.02)	237	0.91 (0.79, 1.04)	193	3.65 (3.10, 4.31)
**Arterial thromboembolism**							
**Female**	baseline	12340	1.00	8104	1.00	1162	1.00
	-28 to -1 days	3247	0.70 (0.67, 0.73)	2141	0.65 (0.62, 0.69)	1281	4.37 (4.01, 4.77)
	0 day	66	0.38 (0.30, 0.49)	42	0.32 (0.24, 0.43)	424	34.28 (30.53, 38.49)
	1-7 days	1089	0.89 (0.83, 0.95)	860	0.92 (0.86, 1.00)	433	4.90 (4.37, 5.50)
	8-14 days	1201	0.98 (0.93, 1.05)	899	0.94 (0.87, 1.01)	272	3.02 (2.64, 3.46)
	15-21 days	1134	0.97 (0.91, 1.03)	1017	1.05 (0.98, 1.12)	166	1.79 (1.52, 2.11)
	22-28 days	1073	0.97 (0.91, 1.04)	901	0.97 (0.91, 1.05)	123	1.28 (1.06, 1.54)
**Male**	baseline	19594	1.00	13031	1.00	1581	1.00
	-28 to -1 days	5903	0.83 (0.81, 0.86)	3842	0.75 (0.72, 0.78)	1773	4.73 (4.40, 5.09)
	0 day	77	0.30 (0.24, 0.38)	83	0.41 (0.33, 0.52)	741	48.24 (44.02, 52.86)

	1-7 days	1675	0.93 (0.88, 0.98)	1301	0.91 (0.86, 0.97)	857	7.81 (7.16, 8.52)
	8-14 days	1861	1.05 (1.00, 1.10)	1477	1.00 (0.95, 1.06)	645	5.67 (5.16, 6.23)
	15-21 days	1771	1.04 (0.99, 1.09)	1572	1.06 (1.00, 1.12)	260	2.19 (1.91, 2.50)
	22-28 days	1660	1.04 (0.99, 1.10)	1317	0.93 (0.88, 0.99)	155	1.24 (1.05, 1.46)
**Secondary outcomes**							
**Cerebral venous sinus thrombosis (CVST) ***							
**Ischaemic stroke**							
**Female**	baseline	5091	1.00	3171	1.00	550	1.00
	-28 to -1 days	1259	0.64 (0.60, 0.68)	779	0.60 (0.55, 0.65)	555	4.06 (3.57, 4.63)
	0 day	19	0.26 (0.17, 0.41)	11	0.21 (0.12, 0.38)	121	20.43 (16.67, 25.04)
	1-7 days	500	0.98 (0.89, 1.08)	369	0.99 (0.89, 1.11)	149	3.51 (2.91, 4.23)
	8-14 days	528	1.05 (0.96, 1.16)	376	0.99 (0.89, 1.11)	106	2.45 (1.98, 3.03)
	15-21 days	485	1.01 (0.91, 1.11)	444	1.16 (1.05, 1.29)	84	1.90 (1.51, 2.40)
	22-28 days	465	1.03 (0.93, 1.13)	386	1.05 (0.95, 1.18)	55	1.21 (0.92, 1.60)
**Male**	baseline	5264	1.00	3268	1.00	519	1.00
	-28 to -1 days	1411	0.70 (0.65, 0.74)	835	0.63 (0.59, 0.69)	573	4.34 (3.82, 4.94)
	0 day	24	0.33 (0.22, 0.49)	22	0.42 (0.28, 0.64)	152	26.67 (22.13, 32.14)
	1-7 days	468	0.90 (0.81, 0.99)	349	0.93 (0.83, 1.04)	177	4.36 (3.66, 5.20)
	8-14 days	552	1.09 (0.99, 1.19)	413	1.06 (0.95, 1.17)	169	4.06 (3.40, 4.85)
	15-21 days	480	0.99 (0.90, 1.10)	418	1.06 (0.96, 1.18)	90	2.10 (1.67, 2.63)
	22-28 days	455	1.02 (0.92, 1.12)	377	1.00 (0.90, 1.12)	57	1.31 (1.00, 1.73)
**Myocardial infarction**							
**Female**	baseline	7456	1.00	5104	1.00	660	1.00
	-28 to -1 days	2020	0.73 (0.69, 0.77)	1393	0.69 (0.64, 0.73)	747	4.47 (3.99, 5.01)
	0 day	45	0.44 (0.32, 0.58)	33	0.41 (0.29, 0.58)	311	45.07 (39.13, 51.90)
	1-7 days	625	0.85 (0.78, 0.92)	499	0.87 (0.79, 0.96)	287	5.84 (5.06, 6.75)
	8-14 days	689	0.93 (0.86, 1.01)	553	0.93 (0.84, 1.01)	159	3.17 (2.65, 3.78)

	15-21 days	671	0.94 (0.87, 1.02)	594	0.98 (0.89, 1.07)	85	1.62 (1.29, 2.04)
	22-28 days	624	0.92 (0.85, 1.01)	532	0.92 (0.84, 1.01)	66	1.21 (0.94, 1.56)
**Male**	baseline	14613	1.00	10008	1.00	1116	1.00
	-28 to -1 days	4541	0.88 (0.85, 0.91)	3091	0.79 (0.76, 0.83)	1248	4.89 (4.47, 5.34)
	0 day	58	0.31 (0.24, 0.40)	62	0.41 (0.32, 0.53)	595	57.89 (52.11, 64.31)
	1-7 days	1251	0.95 (0.90, 1.01)	984	0.92 (0.86, 0.98)	683	9.29 (8.40, 10.27)
	8-14 days	1336	1.02 (0.97, 1.09)	1114	1.01 (0.94, 1.07)	465	6.07 (5.42, 6.79)
	15-21 days	1328	1.06 (1.00, 1.12)	1194	1.07 (1.00, 1.14)	173	2.14 (1.82, 2.52)
	22-28 days	1264	1.07 (1.01, 1.13)	976	0.92 (0.86, 0.98)	95	1.10 (0.89, 1.36)
**Other rare arterial thrombosis**							
**Female**	baseline	559	1.00	282	1.00	50	1.00
	-28 to -1 days	139	0.61 (0.50, 0.74)	70	0.59 (0.45, 0.78)	53	4.31 (2.83, 6.56)
	0 day	*	0.25 (0.06, 0.99)	0	n/a	19	33.69 (19.46, 58.34)
	1-7 days	37	0.64 (0.46, 0.91)	43	1.20 (0.85, 1.68)	24	5.95 (3.58, 9.89)
	8-14 days	66	1.18 (0.90, 1.56)	33	0.89 (0.61, 1.29)	20	4.75 (2.78, 8.12)
	15-21 days	50	0.96 (0.71, 1.30)	38	1.04 (0.73, 1.48)	11	2.67 (1.37, 5.19)
	22-28 days	57	1.20 (0.90, 1.60)	32	0.92 (0.63, 1.33)	12	2.83 (1.49, 5.35)
**Male**	baseline	910	1.00	450	1.00	89	1.00
	-28 to -1 days	226	0.64 (0.55, 0.75)	113	0.59 (0.47, 0.73)	111	4.94 (3.65, 6.70)
	0 day	*	0.25 (0.08, 0.77)	*	0.13 (0.02, 0.92)	36	36.02 (24.06, 53.93)
	1-7 days	68	0.80 (0.62, 1.04)	50	0.91 (0.68, 1.24)	36	5.00 (3.35, 7.47)
	8-14 days	98	1.22 (0.98, 1.53)	55	0.99 (0.74, 1.32)	45	6.08 (4.19, 8.82)
	15-21 days	77	1.03 (0.81, 1.32)	69	1.25 (0.96, 1.62)	23	3.14 (1.97, 5.02)
	22-28 days	57	0.85 (0.65, 1.13)	70	1.31 (1.01, 1.70)	19	2.55 (1.55, 4.21)

* Model does not converge

Supplementary Table 5: Incidence rate ratios (IRR 95% CI) for celiac disease (negative control) and anaphylaxis (positive control) in pre-defined risk periods immediately before and after exposure to vaccination and before and after a positive SARS-CoV-2 result, adjusted for calendar time from December 1, 2020 to April 24, 2021

		Celiac disease (negative control)		Anaphylaxis (positive control)	
		events	IRR (95% CI)	events	IRR (95% CI)
**Group**	**Time period**				
**ChAdOx1nCoV-19 vaccine**	baseline	2072	1.00	339	1.00
	-28 to -1 days	688	0.95 (0.87, 1.05)	105	0.87 (0.69, 1.10)
	0 day	*	0.08 (0.02, 0.31)	45	10.71 (7.72, 14.85)
	1-7 days	157	0.88 (0.74, 1.04)	49	1.71 (1.24, 2.35)
	8-14 days	180	1.03 (0.88, 1.21)	23	0.83 (0.53, 1.28)
	15-21 days	158	0.96 (0.81, 1.14)	28	1.04 (0.70, 1.56)
	22-28 days	<155	1.00 (0.84, 1.19)	23	0.96 (0.62, 1.48)
**BNT162b2 mRNA vaccine**	baseline	1280	1.00	110	1.00
	-28 to -1 days	428	0.88 (0.78, 0.98)	30	0.73 (0.48, 1.11)
	0 day	4	0.22 (0.08, 0.58)	29	18.87 (12.37, 28.78)
	1-7 days	95	0.74 (0.60, 0.92)	16	1.48 (0.87, 2.53)
	8-14 days	121	0.93 (0.77, 1.13)	21	1.89 (1.17, 3.05)
	15-21 days	118	0.90 (0.74, 1.09)	12	1.11 (0.61, 2.04)
	22-28 days	107	0.85 (0.69, 1.04)	10	0.93 (0.48, 1.78)
**SARS-CoV-2**	baseline	173	1.00	43	1.00
	-28 to -1 days	83	1.98 (1.50, 2.61)	14	1.55 (0.81, 2.97)
	0 day	41	25.94 (18.26, 36.84)	9	25.82 (12.16, 54.80)
	1-7 days	66	5.85 (4.36, 7.86)	8	3.23 (1.48, 7.06)
	8-14 days	51	4.29 (3.11, 5.92)	*	0.41 (0.06, 2.97)
	15-21 days	15	1.19 (0.70, 2.03)	*	0.38 (0.05, 2.77)
	22-28 days	19	1.40 (0.87, 2.25)	5	1.73 (0.68, 4.43)

Supplementary table 6a: Background crude incidence rates per 100,000 person years (95% CI) of primary outcomes in people aged 16+ for the 5 calendar years 2015-2019 using primary care data from the QResearch database (representative 20% sample of England) linked to hospital and mortality records

	Thrombocytopenia			Venous thromboembolism			Arterial thromboembolism		
	count	pyrs (100,000)	Rate (95% CI)	count	Pyrs (100,000)	Rate (95% CI)	count	Pyrs (100,000)	Rate (95% CI)
total	16478	440,71	37.39 (36.82 to 37.97)	80444	435,03	184.92 (183.64 to 186.20)	145075	430,12	337.29 (335.56 to 339.03)
Sex									
female	7308	221,23	33.03 (32.28 to 33.80)	42615	218,00	195.48 (193.63 to 197.34)	63576	217,71	292.02 (289.76 to 294.30)
male	9170	219,48	41.78 (40.93 to 42.65)	37829	217,02	174.31 (172.56 to 176.07)	81499	212,41	383.69 (381.07 to 386.33)
Age									
16-19	272	24,04	11.31 (10.05 to 12.74)	540	24,06	22.44 (20.63 to 24.42)	62	24,08	2.58 (2.01 to 3.30)
20-24	507	37,54	13.50 (12.38 to 14.73)	1393	37,54	37.11 (35.21 to 39.11)	210	37,60	5.59 (4.88 to 6.39)
25-29	717	43,69	16.41 (15.25 to 17.66)	2168	43,62	49.70 (47.65 to 51.84)	433	43,75	9.90 (9.01 to 10.87)
30-34	788	43,23	18.23 (17.00 to 19.54)	2781	43,11	64.52 (62.16 to 66.96)	898	43,29	20.74 (19.43 to 22.14)
35-39	801	39,34	20.36 (19.00 to 21.82)	2948	39,15	75.31 (72.64 to 78.07)	1704	39,37	43.28 (41.28 to 45.39)
40-44	851	37,26	22.84 (21.35 to 24.42)	3663	36,99	99.02 (95.87 to 102.28)	3398	37,20	91.36 (88.34 to 94.48)
45-49	1011	38,71	26.12 (24.56 to 27.78)	4902	38,30	127.98 (124.45 to 131.61)	6744	38,42	175.53 (171.39 to 179.76)
50-54	1219	37,28	32.70 (30.92 to 34.59)	5911	36,80	160.61 (156.56 to 164.75)	9803	36,69	267.19 (261.96 to 272.53)
55-59	1363	31,65	43.07 (40.84 to 45.42)	6118	31,16	196.33 (191.47 to 201.31)	11861	30,76	385.66 (378.78 to 392.66)
60-64	1464	26,45	55.34 (52.58 to 58.25)	6996	25,92	269.87 (263.62 to 276.27)	12934	25,34	510.51 (501.79 to 519.38)
65-69	1754	26,20	66.95 (63.89 to 70.15)	9225	25,51	361.57 (354.27 to 369.03)	16167	24,68	655.08 (645.05 to 665.25)
70-74	1671	19,86	84.15 (80.21 to 88.29)	8925	19,18	465.22 (455.67 to 474.97)	16807	18,26	920.27 (906.46 to 934.29)
75-79	1644	15,09	108.93 (103.79 to 114.32)	8718	14,45	603.46 (590.93 to 616.26)	18461	13,44	1373.57 (1353.90 to 1393.53)
80-84	1297	10,70	121.26 (114.84 to 128.04)	7647	10,15	753.62 (736.92 to 770.70)	18881	9,21	2049.25 (2020.23 to 2078.69)

85-89	746	6,30	118.32 (110.13 to 127.13)	5402	5,92	912.08 (888.08 to 936.73)	15394	5,28	2917.83 (2872.10 to 2964.29)
90-94	307	2,64	116.37 (104.05 to 130.14)	2472	2,47	1001.90 (963.17 to 1042.18)	8578	2,17	3956.02 (3873.18 to 4040.63)
95+	56	0,64	87.33 (67.21 to 113.48)	581	0,60	967.91 (892.32 to 1049.90)	2417	0,52	4610.73 (4430.53 to 4798.25)
Ethnicity									
White	11022	273,4	40.31 (39.57 to 41.07)	57084	269,14	212.10 (210.37 to 213.85)	100015	265,73	376.38 (374.06 to 378.72)
Indian	410	11,65	35.19 (31.95 to 38.77)	1084	11,58	93.58 (88.17 to 99.32)	2819	11,44	246.32 (237.39 to 255.58)
Pakistani	282	8,08	34.91 (31.07 to 39.23)	748	8,05	92.96 (86.53 to 99.87)	2335	7,89	295.76 (284.01 to 308.01)
Bangladeshi	197	5,60	35.18 (30.59 to 40.45)	344	5,60	61.47 (55.30 to 68.32)	1298	5,51	235.65 (223.17 to 248.83)
Other Asian	230	7,61	30.22 (26.56 to 34.39)	469	7,60	61.75 (56.41 to 67.60)	1191	7,53	158.15 (149.42 to 167.39)
Caribbean	295	5,15	57.24 (51.06 to 64.15)	1334	5,07	263.25 (249.49 to 277.76)	1796	5,05	355.48 (339.41 to 372.31)
Black African	598	9,98	59.94 (55.32 to 64.94)	1184	9,95	118.99 (112.40 to 125.96)	1245	9,96	125.05 (118.29 to 132.19)
Chinese	75	3,89	19.27 (15.37 to 24.17)	96	3,89	24.65 (20.18 to 30.11)	218	3,89	56.09 (49.12 to 64.06)
Other	495	14,65	33.79 (30.94 to 36.90)	1524	14,58	104.56 (99.44 to 109.94)	1951	14,55	134.06 (128.24 to 140.14)
Not recorded	2874	100,69	28.54 (27.52 to 29.61)	16577	99,58	166.47 (163.96 to 169.03)	32207	98,57	326.75 (323.20 to 330.34)
Townsend									
Quintile 1 (least deprived)	3998	103,66	38.57 (37.39 to 39.78)	20766	102,16	203.26 (200.52 to 206.05)	38528	100,85	382.02 (378.22 to 385.85)
2	3625	96,46	37.58 (36.38 to 38.82)	18968	95,04	199.58 (196.76 to 202.44)	34916	93,89	371.89 (368.01 to 375.81)
3	3094	86,66	35.70 (34.47 to 36.98)	16378	85,47	191.63 (188.72 to 194.59)	29815	84,44	353.07 (349.09 to 357.10)
4	2929	79,34	36.92 (35.60 to 38.28)	13454	78,42	171.57 (168.70 to 174.50)	24217	77,55	312.28 (308.37 to 316.24)
Quintile 5 (most deprived)	2787	73,30	38.02 (36.63 to 39.46)	10712	72,67	147.41 (144.64 to 150.23)	17352	72,11	240.62 (237.06 to 244.22)
Not recorded	45	1,28	35.06 (26.18 to 46.96)	166	1,27	130.45 (112.04 to 151.88)	247	1,27	194.78 (171.95 to 220.65)

Supplementary table 6b: Background crude incidence rates per 100,000 person years (95% CI) of secondary outcomes in people aged 16+ for the 5 calendar years 2015-2019 using primary care data from the QResearch database (representative 20% sample of England) linked to hospital and mortality records.

	Cerebral Venous Sinus Thrombosis (CVST)		Ischaemic stroke		Myocardial infarction		Other rare arterial thrombotic event	
	**count**	**Rate (95% CI)**	**count**	**Rate (95% CI)**	**count**	**Rate (95% CI)**	**count**	**Rate (95% CI)**
total	559	1.26 (1.16 to 1.37)	108707	251.24 (249.75 to 252.74)	76546	176.04 (174.80 to 177.29)	8369	18.94 (18.54 to 19.35)
Sex								
female	346	1.56 (1.40 to 1.73)	54351	249.97 (247.88 to 252.08)	28099	127.75 (126.27 to 129.25)	3690	16.63 (16.10 to 17.18)
male	213	0.97 (0.85 to 1.11)	54356	252.52 (250.41 to 254.65)	48447	225.47 (223.48 to 227.49)	4679	21.27 (20.67 to 21.89)
Age								
16-19	29	1.20 (0.84 to 1.73)	79	3.28 (2.63 to 4.09)	10	0.42 (0.22 to 0.77)	17	0.71 (0.44 to 1.14)
20-24	54	1.44 (1.10 to 1.87)	239	6.36 (5.60 to 7.22)	59	1.57 (1.22 to 2.02)	34	0.90 (0.65 to 1.27)
25-29	50	1.14 (0.87 to 1.51)	407	9.30 (8.44 to 10.25)	153	3.50 (2.98 to 4.10)	45	1.03 (0.77 to 1.38)
30-34	54	1.25 (0.95 to 1.63)	733	16.93 (15.75 to 18.20)	417	9.62 (8.74 to 10.59)	103	2.38 (1.96 to 2.88)
35-39	48	1.22 (0.92 to 1.61)	1180	29.96 (28.30 to 31.72)	971	24.64 (23.13 to 26.23)	123	3.12 (2.61 to 3.72)
40-44	50	1.34 (1.01 to 1.76)	2066	55.45 (53.11 to 57.89)	2142	57.46 (55.08 to 59.94)	213	5.70 (4.98 to 6.52)
45-49	64	1.65 (1.29 to 2.11)	3879	100.55 (97.44 to 103.77)	4346	112.65 (109.35 to 116.04)	409	10.54 (9.56 to 11.61)
50-54	42	1.12 (0.83 to 1.52)	6014	162.66 (158.60 to 166.82)	6307	170.70 (166.54 to 174.97)	598	16.00 (14.76 to 17.33)
55-59	32	1.01 (0.71 to 1.42)	7555	242.47 (237.06 to 248.00)	7504	241.30 (235.90 to 246.82)	735	23.17 (21.55 to 24.90)
60-64	42	1.58 (1.17 to 2.14)	9049	351.21 (344.05 to 358.52)	7750	300.85 (294.22 to 307.62)	895	33.75 (31.61 to 36.04)
65-69	25	0.95 (0.64 to 1.40)	12411	493.02 (484.42 to 501.77)	9116	360.83 (353.50 to 368.32)	1050	39.98 (37.63 to 42.47)
70-74	26	1.30 (0.88 to 1.91)	13820	741.64 (729.37 to 754.10)	8905	471.14 (461.45 to 481.03)	1088	54.66 (51.50 to 58.00)
75-79	19	1.25 (0.80 to 1.96)	15689	1145.75 (1127.97 to 1163.82)	8884	628.87 (615.93 to 642.08)	1080	71.32 (67.19 to 75.70)
80-84	11	1.02 (0.56 to 1.84)	15344	1644.05 (1618.24 to 1670.27)	8633	875.87 (857.59 to 894.55)	921	85.82 (80.46 to 91.55)

85-89	10	1.57 (0.85 to 2.92)	11881	2247.04 (2207.00 to 2287.81)	6791	1182.66 (1154.86 to 1211.12)	672	106.31 (98.57 to 114.66)
90-94	*	1.13 (0.36 to 3.49)	6356	2959.24 (2887.38 to 3032.89)	3512	1463.83 (1416.21 to 1513.05)	296	112.06 (100.00 to 125.58)
95+	*	n/a	1773	3432.20 (3276.10 to 3595.74)	937	1611.09 (1511.16 to 1717.62)	75	116.95 (93.27 to 146.66)
Ethnicity								
White	340	1.24 (1.11 to 1.38)	76319	285.27 (283.25 to 287.30)	52295	194.36 (192.71 to 196.04)	6155	22.46 (21.90 to 23.03)
Indian	21	1.80 (1.17 to 2.75)	1697	146.99 (140.16 to 154.15)	1886	163.93 (156.70 to 171.50)	91	7.79 (6.34 to 9.56)
Pakistani	20	2.47 (1.59 to 3.82)	1289	161.27 (152.70 to 170.32)	1694	213.18 (203.27 to 223.58)	70	8.64 (6.83 to 10.92)
Bangladeshi	*	0.53 (0.17 to 1.66)	689	123.95 (115.03 to 133.56)	939	169.65 (159.14 to 180.85)	34	6.05 (4.33 to 8.47)
Other Asian	10	1.31 (0.70 to 2.43)	754	99.63 (92.76 to 107.00)	765	101.19 (94.27 to 108.62)	48	6.29 (4.74 to 8.35)
Caribbean	11	2.12 (1.17 to 3.83)	1487	294.65 (280.05 to 310.02)	743	144.93 (134.88 to 155.74)	124	23.94 (20.08 to 28.55)
Black African	18	1.79 (1.13 to 2.85)	1187	119.39 (112.79 to 126.38)	492	49.17 (45.01 to 53.71)	85	8.47 (6.85 to 10.48)
Chinese	*	0.26 (0.04 to 1.82)	173	44.52 (38.36 to 51.67)	112	28.77 (23.91 to 34.62)	7	1.79 (0.86 to 3.76)
Other	22	1.50 (0.99 to 2.27)	1418	97.26 (92.33 to 102.46)	1084	74.18 (69.89 to 78.73)	114	7.76 (6.46 to 9.32)
Not recorded	113	1.12 (0.93 to 1.35)	23694	239.26 (236.24 to 242.33)	16536	166.07 (163.55 to 168.62)	1641	16.27 (15.50 to 17.07)
Townsend								
Quintile 1	115	1.10 (0.92 to 1.33)	30653	302.27 (298.91 to 305.67)	19858	194.52 (191.83 to 197.24)	1806	17.37 (16.58 to 18.19)
2	112	1.16 (0.96 to 1.39)	27032	286.14 (282.75 to 289.57)	18154	191.09 (188.33 to 193.89)	1772	18.32 (17.49 to 19.19)
3	115	1.32 (1.10 to 1.59)	21626	254.39 (251.02 to 257.80)	15846	185.53 (182.67 to 188.44)	1766	20.34 (19.41 to 21.31)
4	128	1.61 (1.35 to 1.91)	17270	221.28 (218.01 to 224.61)	13063	166.74 (163.90 to 169.62)	1619	20.37 (19.40 to 21.38)
Quintile 5	88	1.20 (0.97 to 1.47)	11924	164.52 (161.60 to 167.50)	9501	130.70 (128.10 to 133.35)	1385	18.85 (17.88 to 19.87)
Not recorded	*	0.78 (0.11 to 5.51)	202	158.87 (138.40 to 182.36)	124	97.13 (81.45 to 115.82)	21	16.31 (10.63 to 25.02)

Supplementary table 7a: Incidence rate ratios (IRR 95% CI) for primary composite outcomes in pre-defined risk periods immediately before and after exposure to vaccination, adjusted for calendar time from December 1, 2020 to April 24, 2021. Comparisons between different sensitivity analyses.

		Sensitivity 1*		Sensitivity 2*		Sensitivity 3*		Sensitivity 4*		Sensitivity 5*		Sensitivity 6*	
		events	IRR (95% CI)	events	IRR (95% CI)	events	IRR (95% CI)	events	IRR (95% CI)	events	IRR (95% CI)	events	IRR (95% CI)
**Thrombocytopenia**													
**ChAdOx1nCoV-19 vaccine**	baseline	3840	1.00	1106	1.00	3849	1.00	2732	1.00	3563	1.00	3851	1.00
	-28 to -1 days	909	0.67 (0.62, 0.72)			910	0.67 (0.62, 0.72)	836	0.65 (0.60, 0.71)	899	0.67 (0.62, 0.72)	910	0.66 (0.61, 0.71)
	0 day	19	0.40 (0.25, 0.62)	19	0.37 (0.23, 0.59)	19	0.39 (0.25, 0.62)	19	0.46 (0.29, 0.72)	19	0.39 (0.25, 0.62)	19	0.39 (0.25, 0.61)
	1-7 days	331	0.98 (0.87, 1.10)	331	0.91 (0.76, 1.09)	331	0.97 (0.87, 1.10)	270	0.94 (0.82, 1.08)	320	0.98 (0.87, 1.10)	331	0.97 (0.86, 1.09)
	8-14 days	425	1.30 (1.16, 1.44)	438	1.26 (1.08, 1.47)	438	1.32 (1.19, 1.47)	324	1.23 (1.08, 1.41)	410	1.33 (1.19, 1.47)	438	1.32 (1.18, 1.47)
	15-21 days	332	1.08 (0.96, 1.22)	337	1.06 (0.91, 1.23)	337	1.08 (0.96, 1.22)	238	1.00 (0.86, 1.16)	304	1.09 (0.97, 1.22)	337	1.08 (0.96, 1.21)
	22-28 days	354	1.27 (1.13, 1.42)	356	1.26 (1.10, 1.44)	356	1.26 (1.12, 1.41)	240	1.19 (1.03, 1.38)	314	1.27 (1.13, 1.42)	356	1.26 (1.12, 1.41)
**BNT162b2 mRNA vaccine**	baseline	1993	1.00	1157	1.00	1997	1.00	1372	1.00	1871	1.00	1862	1.00
	-28 to -1 days	504	0.63 (0.57, 0.70)			504	0.62 (0.56, 0.69)	499	0.62 (0.56, 0.69)	503	0.63 (0.56, 0.69)	461	0.62 (0.56, 0.69)
	0 day	13	0.41 (0.24, 0.71)	13	0.33 (0.18, 0.59)	13	0.40 (0.23, 0.69)	13	0.41 (0.23, 0.70)	13	0.41 (0.23, 0.70)	12	0.42 (0.24, 0.74)
	1-7 days	240	1.02 (0.89, 1.18)	243	0.86 (0.69, 1.06)	243	1.01 (0.88, 1.16)	239	1.00 (0.86, 1.15)	243	1.02 (0.89, 1.18)	204	0.95 (0.82, 1.11)
	8-14 days	253	1.03 (0.90, 1.18)	254	0.92 (0.76, 1.11)	254	1.01 (0.88, 1.16)	251	1.00 (0.87, 1.16)	253	1.02 (0.89, 1.17)	214	0.98 (0.84, 1.13)
	15-21 days	258	1.07 (0.94, 1.23)	259	1.02 (0.86, 1.21)	259	1.05 (0.92, 1.20)	240	1.04 (0.90, 1.20)	259	1.06 (0.93, 1.22)	217	1.02 (0.88, 1.18)
	22-28 days	241	1.09 (0.95, 1.25)	241	1.05 (0.89, 1.23)	241	1.06 (0.92, 1.22)	205	1.03 (0.89, 1.20)	239	1.08 (0.94, 1.23)	215	1.06 (0.91, 1.22)

**SARS-CoV-2 positive test**	baseline	380	1.00	378	1.00					354	1.00		
	-28 to -1 days	427	4.67 (4.02, 5.43)							428	4.72 (4.06, 5.48)		
	0 day	298	75.50 (64.25, 88.73)	564	160.48 (134.56, 192, 38)					297	75.75 (64.48, 89.00)		
	1-7 days	395	13.94 (11.99, 16.20)	494	19.8 (16,68, 23,66)					398	14.04 (12.09, 16.32)		
	8-14 days	149	5.18 (4.26, 6.29)	183	7.23 (5.90, 8.87)					151	5.27 (4.34, 6.40)		
	15-21 days	56	1.91 (1.44, 2.54)	81	3.16 (2.44, 4.08)					56	1.91 (1.44, 2.54)		
	22-28 days	45	1.50 (1.10, 2.05)	54	2.10 (1.59, 2.82)					45	1.50 (1.10, 2.05)		
**Venous thromboembolism**													
**ChAdOx1nCoV-19 vaccine**	baseline	9314	1.00	2320	1.00	9837	1.00	7488	1.00	9222	1.00	9846	1.00
	-28 to -1 days	2520	0.74 (0.70, 0.78)			2561	0.70 (0.66, 0.73)	2401	0.69 (0.66, 0.73)	2548	0.70 (0.66, 0.73)	2561	0.69 (0.66, 0.72)
	0 day	46	0.45 (0.33, 0.60)	53	0.46 (0.35, 0.62)	53	0.46 (0.35, 0.60)	46	0.47 (0.35, 0.63)	52	0.46 (0.35, 0.60)	53	0.45 (0.35, 0.59)
	1-7 days	613	0.85 (0.78, 0.93)	746	0.92 (0.82, 1.04)	746	0.92 (0.85, 1.00)	638	0.96 (0.87, 1.04)	736	0.92 (0.85, 1.00)	746	0.91 (0.84, 0.98)
	8-14 days	742	1.07 (0.99, 1.16)	870	1.12 (1.00, 1.24)	870	1.10 (1.02, 1.18)	663	1.09 (0.99, 1.19)	830	1.10 (1.02, 1.18)	870	1.08 (1.01, 1.17)
	15-21 days	634	0.99 (0.91, 1.08)	763	1.08 (0.97, 1.19)	763	1.03 (0.95, 1.12)	560	1.03 (0.93, 1.13)	702	1.04 (0.96, 1.12)	763	1.02 (0.94, 1.11)
	22-28 days	536	0.94 (0.86, 1.03)	645	1.02 (0.93, 1.13)	645	0.97 (0.89, 1.06)	451	1.01 (0.91, 1.12)	576	0.98 (0.90, 1.06)	645	0.96 (0.88, 1.05)
**BNT162b2 mRNA vaccine**	baseline	4202	1.00	2388	1.00	4584	1.00	3322	1.00	4315	1.00	4275	1.00
	-28 to -1 days	1197	0.66 (0.62, 0.71)			1224	0.63 (0.59, 0.68)	1199	0.62 (0.58, 0.67)	1220	0.64 (0.59, 0.68)	1157	0.63 (0.59, 0.68)
	0 day	24	0.37	25	0.32	25	0.35	25	0.35	25	0.35	22	0.33

			(0.25, 0.55)		(0.21, 0.48)		(0.23, 0.52)		(0.24, 0.52)		(0.24, 0.52)		(0.21, 0.50)
	1-7 days	405	0.84 (0.76, 0.93)	486	0.84 (0.72, 0.97)	486	0.91 (0.83, 1.01)	477	0.91 (0.82, 1.00)	482	0.92 (0.83, 1.01)	415	0.83 (0.75, 0.92)
	8-14 days	464	0.92 (0.83, 1.02)	555	0.96 (0.84, 1.09)	555	0.98 (0.90, 1.08)	544	1.00 (0.91, 1.10)	553	0.99 (0.90, 1.08)	477	0.92 (0.83, 1.01)
	15-21 days	429	0.85 (0.77, 0.95)	514	0.94 (0.83, 1.06)	514	0.90 (0.82, 0.99)	479	0.94 (0.85, 1.04)	510	0.91 (0.82, 1.00)	425	0.82 (0.74, 0.91)
	22-28 days	393	0.85 (0.76, 0.94)	474	0.95 (0.85, 1.07)	474	0.89 (0.80, 0.98)	408	0.92 (0.82, 1.02)	473	0.89 (0.81, 0.98)	419	0.87 (0.78, 0.96)
**SARS-CoV-2 positive test**	baseline	1088	1.00	1230	1.00					1137	1.00		
	-28 to -1 days	946	3.37 (3.07, 3.70)							1002	3.44 (3.14, 3.77)		
	0 day	788	63.97 (58.01, 70.55)	1510	84.42 (76.15, 93.56)					826	63.57 (57.85, 69.85)		
	1-7 days	1232	13.86 (12.69, 15.13)	1707	13.59 (12.31, 14.99)					1303	13.79 (12.67, 15.00)		
	8-14 days	1346	14.49 (13.30, 15.78)	1709	13.53 (12.33, 14.86)					1366	13.87 (12.77, 15.07)		
	15-21 days	791	8.22 (7.47, 9.04)	1019	8.12 (7.37, 8.95)					802	7.88 (7.19, 8.65)		
	22-28 days	345	3.50 (3.09, 3.95)	444	3.62 (3.22, 4.07)								
**Arterial thromboembolism**													
**ChAdOx1nCoV-19 vaccine**	Baseline	29999	1.00	11409	1.00	31895	1.00	22075	1.00	28942	1.00	31944	1.00
	-28 to -1 days	9022	0.84 (0.82, 0.86)			9155	0.78 (0.77, 0.80)	8574	0.78 (0.76, 0.80)	9083	0.78 (0.76, 0.80)	9155	0.78 (0.76, 0.80)
	0 day	115	0.30 (0.25, 0.36)	143	0.31 (0.26, 0.37)	143	0.33 (0.28, 0.39)	130	0.35 (0.29, 0.42)	143	0.33 (0.28, 0.39)	143	0.33 (0.28, 0.39)
	1-7 days	2397	0.88 (0.84, 0.92)	2769	0.86 (0.81, 0.91)	2769	0.92 (0.88, 0.96)	2300	0.90 (0.86, 0.95)	2714	0.92 (0.88, 0.95)	2769	0.91 (0.87, 0.94)
	8-14 days	2647	0.99 (0.95, 1.03)	3065	0.98 (0.93, 1.04)	3065	1.03 (0.99, 1.07)	2445	1.04 (0.99, 1.09)	2942	1.02 (0.99, 1.07)	3065	1.01 (0.97, 1.05)

	15-21 days	2485	0.97 (0.93, 1.01)	2906	0.99 (0.94, 1.04)	2906	1.01 (0.97, 1.05)	2228	1.04 (0.99, 1.09)	2691	1.01 (0.97, 1.06)	2906	1.00 (0.96, 1.04)
	22-28 days	2385	1.00 (0.96, 1.04)	2734	1.00 (0.95, 1.05)	2734	1.01 (0.97, 1.06)	1895	1.03 (0.98, 1.09)	2479	1.02 (0.98, 1.06)	2734	1.01 (0.97, 1.05)
**BNT162b2 mRNA vaccine**	baseline	19482	1.00	12649	1.00	21009	1.00	14606	1.00	19477	1.00	19399	1.00
	-28 to -1 days	5925	0.74 (0.72, 0.77)			5984	0.70 (0.68, 0.73)	5917	0.70 (0.68, 0.72)	5973	0.71 (0.69, 0.73)	5277	0.70 (0.68, 0.73)
	0 day	110	0.36 (0.30, 0.43)	125	0.32 (0.27, 0.38)	125	0.37 (0.31, 0.45)	125	0.38 (0.32, 0.45)	125	0.38 (0.32, 0.45)	105	0.36 (0.30, 0.44)
	1-7 days	1894	0.87 (0.83, 0.92)	2161	0.81 (0.75, 0.86)	2161	0.91 (0.87, 0.95)	2148	0.91 (0.87, 0.95)	2156	0.92 (0.88, 0.96)	1785	0.86 (0.82, 0.91)
	8-14 days	2083	0.94 (0.90, 0.98)	2377	0.92 (0.86, 0.97)	2377	0.97 (0.93, 1.01)	2320	0.97 (0.93, 1.01)	2372	0.98 (0.94, 1.02)	1988	0.93 (0.89, 0.98)
	15-21 days	2284	1.03 (0.98, 1.07)	2590	1.03 (0.98, 1.09)	2590	1.05 (1.00, 1.09)	2393	1.08 (1.03, 1.13)	2583	1.06 (1.01, 1.10)	2154	1.00 (0.95, 1.05)
	22-28 days	1961	0.93 (0.88, 0.97)	2221	0.95 (0.90, 1.00)	2221	0.94 (0.90, 0.98)	1883	0.98 (0.93, 1.03)	2211	0.95 (0.91, 0.99)	1956	0.92 (0.88, 0.96)
**SARS-CoV-2 positive test**	baseline	2376	1.00	2225	1.00					2508	1.00		
	-28 to -1 days	2913	4.86 (4.59, 5.16)							3044	4.60 (4.35, 4.87)		
	0 day	1096	45.15 (41.89, 48.67)	2300	97.66 (90.27, 105.66)					1160	42.33 (39.40, 45.47)		
	1-7 days	1138	6.58 (6.11, 7.09)	1630	9.85 (9.07, 10.68)					1288	6.56 (6.13, 7.03)		
	8-14 days	798	4.48 (4.13, 4.87)	978	5.86 (5.36, 6.39)					913	4.53 (4.19, 4.89)		
	15-21 days	361	1.94 (1.74, 2.17)	459	2.72 (2.43, 3.04)					422	2.02 (1.82, 2.24)		
	22-28 days	242	1.24 (1.09, 1.42)	295	1.74 (1.53, 1.98)					273	1.26 (1.12, 1.43)		

*Sensitivity 1: excluding those who died from the outcome; Sensitivity 2: restricting analysis to the period post-vaccination, without censoring at death (restricting analysis to the period post-SARS-CoV-2 positive test, without censoring at death); Sensitivity 3: censoring at 12 weeks post vaccination, Sensitivity 4: censoring on 10 March 2021; Sensitivity 5: restricting the study period until the 31 March 2021; Sensitivity 6: dropping those who received vaccination before 1 January 2021.

Supplementary table 7b: Incidence rate ratios (IRR 95% CI) for secondary outcomes in pre-defined risk periods immediately before and after exposure to vaccination, adjusted for calendar time from December 1, 2020 to April 24, 2021. Comparisons between different sensitivity analyses. (see footnote table 5a for description of sensitivity analyses)

		Sensitivity 1*		Sensitivity 2*		Sensitivity 3*		Sensitivity 4*		Sensitivity 5*		Sensitivity 6*	
		events	IRR (95% CI)	events	IRR (95% CI)	events	IRR (95% CI)	events	IRR (95% CI)	events	IRR (95% CI)	events	IRR (95% CI)
**Cerebral venous sinus thrombosis**													
**ChAdOx1nCoV-19 vaccine**	baseline	44	1.00	13	1.00	44	1.00	32	1.00	41	1.00	44	1.00
	-28 to -1 days	9	0.59 (0.27, 1.25)			9	0.59 (0.28, 1.25)	7	0.51 (0.21, 1.26)	9	0.59 (0.27, 1.25)	9	0.59 (0.28, 1.27)
	0 day	*	1.95 (0.26, 14.48)	*	0.70 (0.07, 7.55)	*	1.94 (0.26, 14.38)	*	3.23 (0.41, 25.52)	*	1.96 (0.26, 14.49)	*	1.97 (0.26, 14.61)
	1-7 days	*	0.51 (0.12, 2.19)	*	0.23 (0.04, 1.46)	*	0.51 (0.12, 2.16)	*	0.46 (0.06, 3.73)	*	0.52 (0.12, 2.19)	*	0.51 (0.12, 2.19)
	8-14 days	15	4.01 (2.08, 7.71)	15	1.94 (0.57, 6.60)	15	3.94 (2.05, 7.56)	5	2.94 (0.92, 9.45)	11	4.01 (2.08, 7.70)	15	3.95 (2.05, 7.64)
	15-21 days	8	2.15 (0.96, 4.85)	8	1.27 (0.40, 4.07)	8	2.10 (0.94, 4.73)	*	1.87 (0.46, 7.67)	5	2.16 (0.96, 4.87)	8	2.13 (0.94, 4.81)
	22-28 days	*	0.60 (0.14, 2.55)	*	0.38 (0.08, 1.87)	*	0.58 (0.14, 2.48)	*	n/a	*	0.60 (0.14, 2.58)	*	0.59 (0.14, 2.51)
**BNT162b2 mRNA vaccine**	baseline	21	1.00	10	1.00	21	1.00	15	1.00	20	1.00	18	1.00
	-28 to -1 days	7	1.04 (0.43, 2.51)			7	0.99 (0.41, 2.40)	7	0.81 (0.32, 2.04)	7	1.04 (0.43, 2.52)	6	1.05 (0.40, 2.78)
	0 day	*	n/a	*	n/a	*	n/a	*	n/a				
	1-7 days	*	n/a	*	n/a	*	n/a	*	n/a				
	8-14 days	*	2.57 (0.85, 7.78)	*	1.43 (0.27, 7.47)	*	2.45 (0.81, 7.40)	*	1.86 (0.51, 6.84)	*	2.56 (0.85, 7.76)	*	2.95 (0.94, 9.24)
	15-21 days	6	3.58 (1.39, 9.27)	6	2.37 (0.57, 9.74)	6	3.38 (1.31, 8.72)	*	2.75 (0.83, 9.07)	5	3.59 (1.39, 9.27)	5	3.60 (1.25, 10.33)
	22-28 days	*	n/a	*	n/a	*	n/a	*	n/a				
**SARS-CoV-2 positive test**	baseline	*	1.00	*	1.00					*	1.00		
	-28 to -1 days	*	2.40 (0.22, 25.98)	*						*	2.40 (0.22, 25.93)		

	0 day	*	115.70 (16.48, 812.29)	*	173.30 (18.75, 1601.53)					*	115.46 (16.44, 810.75)		
	1-7 days	*	12.90 (1.86, 89.64)	*	5.70 (0.35, 91.90)					*	12.88 (1.85, 89.49)		
	8-14 days	*	13.43 (1.99, 90.59)	*	20.46 (2.28, 183.68)					*	13.40 (1.99, 90.40)		
	15-21 days	*	6.33 (0.63, 63.67)	*	27.30 (3.36, 224.97)					*	6.32 (0.63, 63.54)		
	22-28 days	*	5.81 (0.59, 57.24)	*	6.56 (0.49, 86.78)					*	5.80 (0.59, 57.15)		
**Ischaemic Stroke**													
**ChAdOx1nCoV-19 vaccine**	baseline	8995	1.00	3937	1.00	10320	1.00	7028	1.00	9371	1.00	10355	1.00
	-28 to -1 days	2578	0.78 (0.74, 0.82)			2671	0.67 (0.64, 0.71)	2496	0.68 (0.65, 0.71)	2647	0.67 (0.64, 0.70)	2671	0.67 (0.64, 0.70)
	0 day	27	0.23 (0.16, 0.34)	43	0.26 (0.19, 0.36)	43	0.30 (0.22, 0.40)	38	0.30 (0.22, 0.42)	43	0.29 (0.22, 0.40)	43	0.29 (0.21, 0.39)
	1-7 days	751	0.92 (0.85, 0.99)	968	0.86 (0.78, 0.95)	968	0.95 (0.88, 1.01)	838	0.98 (0.91, 1.06)	948	0.94 (0.88, 1.01)	968	0.93 (0.87, 1.00)
	8-14 days	800	1.01 (0.94, 1.09)	1080	1.01 (0.92, 1.10)	1080	1.07 (1.01, 1.15)	878	1.11 (1.03, 1.20)	1039	1.07 (1.00, 1.15)	1080	1.06 (0.99, 1.13)
	15-21 days	689	0.92 (0.85, 1.00)	965	0.96 (0.88, 1.04)	965	1.00 (0.93, 1.07)	768	1.06 (0.98, 1.15)	904	1.00 (0.94, 1.07)	965	0.99 (0.92, 1.06)
	22-28 days	671	0.97 (0.89, 1.05)	920	0.99 (0.91, 1.07)	920	1.02 (0.95, 1.10)	673	1.07 (0.99, 1.17)	857	1.03 (0.96, 1.10)	920	1.01 (0.94, 1.08)
**BNT162b2 mRNA vaccine**	baseline	5444	1.00	4217	1.00	6374	1.00	4364	1.00	5960	1.00	5778	1.00
	-28 to -1 days	1575	0.67 (0.63, 0.71)			1614	0.61 (0.58, 0.65)	1595	0.61 (0.58, 0.65)	1611	0.62 (0.58, 0.65)	1428	0.62 (0.58, 0.66)
	0 day	28	0.31 (0.22, 0.45)	33	0.24 (0.17, 0.34)	33	0.31 (0.22, 0.44)	33	0.32 (0.23, 0.45)	33	0.32 (0.23, 0.45)	26	0.29 (0.20, 0.43)
	1-7 days	579	0.92 (0.84, 1.00)	718	0.77 (0.68, 0.86)	718	0.95 (0.88, 1.03)	713	0.97 (0.89, 1.05)	718	0.97 (0.89, 1.05)	574	0.89 (0.82, 0.98)
	8-14 days	610	0.95	789	0.90	789	1.02	773	1.04	787	1.03	637	0.96

			(0.87, 1.04)		(0.81, 1.00)		(0.94, 1.10)		(0.96, 1.12)		(0.95, 1.11)		(0.88, 1.04)
	15-21 days	671	1.05 (0.97, 1.14)	862	1.03 (0.94, 1.14)	862	1.10 (1.02, 1.19)	812	1.16 (1.07, 1.25)	860	1.12 (1.04, 1.20)	692	1.03 (0.95, 1.12)
	22-28 days	600	0.99 (0.91, 1.08)	765	0.98 (0.90, 1.07)	765	1.02 (0.94, 1.10)	685	1.09 (1.00, 1.19)	765	1.03 (0.96, 1.12)	650	0.97 (0.90, 1.06)
**SARS-CoV-2 positive test**	baseline	772	1.00	788	1.00					977	1.00		
	-28 to -1 days	1030	4.92 (4.45, 5.46)							1123	4.21 (3.85, 4.62)		
	0 day	239	27.75 (23.87, 32.26)	535	62.52 (54.0, 72.34)					273	23.58 (20.56, 27.05)		
	1-7 days	187	3.06 (2.60, 3.61)	298	4.98 (4.23, 5.85)					325	3.94 (3.47, 4.48)		
	8-14 days	163	2.62 (2.21, 3.11)	221	3.69 (3.11, 4.38)					273	3.25 (2.84, 3.73)		
	15-21 days	105	1.63 (1.33, 2.00)	149	2.48 (2.05, 3.00)					171	2.00 (1.70, 2.36)		
	22-28 days	75	1.13 (0.89, 1.44)	92	1.54 (1.22, 1.93)					108	1.26 (1.04, 1.54)		
**Myocardial Infarction**													
**ChAdOx1nCoV-19 vaccine**	baseline	21303	1.00	7863	1.00	22060	1.00	15246	1.00	19954	1.00	22079	1.00
	-28 to -1 days	6524	0.86 (0.84, 0.89)			6565	0.83 (0.81, 0.86)	6159	0.83 (0.80, 0.85)	6515	0.83 (0.81, 0.86)	6565	0.83 (0.80, 0.85)
	0 day	88	0.32 (0.26, 0.40)	103	0.34 (0.28, 0.42)	103	0.36 (0.29, 0.43)	96	0.38 (0.31, 0.46)	103	0.36 (0.29, 0.43)	103	0.35 (0.29, 0.43)
	1-7 days	1704	0.88 (0.84, 0.93)	1881	0.88 (0.82, 0.94)	1881	0.92 (0.88, 0.97)	1544	0.89 (0.84, 0.94)	1846	0.92 (0.87, 0.96)	1881	0.91 (0.87, 0.96)
	8-14 days	1867	0.97 (0.93, 1.02)	2028	0.96 (0.90, 1.03)	2028	0.99 (0.95, 1.04)	1617	1.00 (0.94, 1.06)	1945	0.99 (0.95, 1.04)	2028	0.98 (0.94, 1.03)
	15-21 days	1815	0.98 (0.94, 1.04)	2000	1.00 (0.94, 1.06)	2000	1.02 (0.97, 1.07)	1512	1.02 (0.97, 1.08)	1848	1.02 (0.97, 1.07)	2000	1.01 (0.96, 1.06)
	22-28 days	1747	1.01 (0.96, 1.06)	1889	1.01 (0.95, 1.06)	1889	1.02 (0.97, 1.07)	1288	1.02 (0.96, 1.09)	1699	1.02 (0.97, 1.07)	1889	1.01 (0.96, 1.06)
	baseline	14317	1.00	8784	1.00	15038	1.00	10489	1.00	13891	1.00	13971	1.00

**BNT162b2 mRNA vaccine**	-28 to -1 days	4461	0.78 (0.75, 0.81)			4485	0.75 (0.72, 0.78)	4436	0.74 (0.71, 0.77)	4477	0.76 (0.73, 0.78)	3936	0.75 (0.72, 0.77)
	0 day	84	0.38 (0.31, 0.47)	95	0.37 (0.30, 0.46)	95	0.41 (0.33, 0.50)	95	0.41 (0.33, 0.50)	95	0.41 (0.33, 0.50)	80	0.40 (0.32, 0.49)
	1-7 days	1344	0.86 (0.81, 0.91)	1483	0.83 (0.76, 0.90)	1483	0.89 (0.85, 0.94)	1472	0.88 (0.83, 0.94)	1477	0.90 (0.85, 0.95)	1244	0.86 (0.81, 0.91)
	8-14 days	1532	0.95 (0.90, 1.00)	1668	0.95 (0.88, 1.02)	1668	0.97 (0.92, 1.02)	1627	0.96 (0.91, 1.02)	1665	0.98 (0.93, 1.03)	1409	0.94 (0.89, 0.99)
	15-21 days	1649	1.01 (0.96, 1.07)	1789	1.04 (0.98, 1.11)	1789	1.03 (0.98, 1.08)	1643	1.05 (1.00, 1.11)	1783	1.04 (0.99, 1.09)	1508	0.99 (0.94, 1.05)
	22-28 days	1390	0.90 (0.85, 0.95)	1510	0.94 (0.89, 1.00)	1510	0.91 (0.86, 0.96)	1258	0.93 (0.88, 0.99)	1502	0.92 (0.87, 0.97)	1349	0.90 (0.85, 0.95)
**SARS-CoV-2 positive test**	baseline	1667	1.00	1529	1.00					1616	1.00		
	-28 to -1 days	1942	4.76 (4.44, 5.11)							1989	4.75 (4.43, 5.09)		
	0 day	871	53.34 (48.93, 58.15)	1782	113.74 (103.69, 124.76)					901	53.15 (48.86, 57.83)		
	1-7 days	939	8.06 (7.41, 8.76)	1323	12.01 (10.93, 13.21)					970	7.97 (7.34, 8.65)		
	8-14 days	609	5.05 (4.58, 5.55)	731	6.56 (5.91, 7.27)					622	4.95 (4.50, 5.44)		
	15-21 days	249	1.97 (1.72, 2.25)	303	2.68 (2.34, 3.06)					255	1.95 (1.71, 2.23)		
	22-28 days	156	1.16 (0.99, 1.37)	194	1.70 (1.46, 1.99)					158	1.15 (0.98, 1.35)		
**Other rare arterial thrombotic**													
**ChAdOx1nCoV-19 vaccine**	baseline	1444	1.00	402	1.00	1469	1.00	1087	1.00	1383	1.00	1469	1.00
	-28 to -1 days	363	0.64 (0.56, 0.72)			365	0.63 (0.56, 0.71)	345	0.63 (0.55, 0.72)	363	0.63 (0.56, 0.71)	365	0.62 (0.55, 0.70)
	0 day	5	0.25 (0.10, 0.61)	5	0.27 (0.11, 0.67)	5	0.25 (0.10, 0.59)	*	0.23 (0.09, 0.61)	5	0.25 (0.10, 0.59)	5	0.24 (0.10, 0.58)
	1-7 days	102	0.74	105	0.81	105	0.74	89	0.74	103	0.74	105	0.73

			(0.60, 0.91)		(0.60, 1.10)		(0.60, 0.91)		(0.59, 0.94)		(0.60, 0.91)		(0.59, 0.89)
	8-14 days	154	1.17 (0.98, 1.40)	164	1.32 (1.02, 1.70)	164	1.21 (1.02, 1.43)	137	1.27 (1.03, 1.55)	160	1.21 (1.02, 1.44)	164	1.19 (1.00, 1.41)
	15-21 days	123	1.00 (0.82, 1.21)	127	1.09 (0.85, 1.39)	127	1.00 (0.82, 1.21)	100	1.02 (0.81, 1.28)	117	1.00 (0.83, 1.21)	127	0.99 (0.81, 1.19)
	22-28 days	111	1.00 (0.82, 1.23)	114	1.08 (0.85, 1.36)	114	0.99 (0.81, 1.21)	87	1.05 (0.83, 1.34)	106	1.00 (0.82, 1.22)	114	0.99 (0.81, 1.20)
**BNT162b2 mRNA vaccine**	baseline	711	1.00	444	1.00	727	1.00	499	1.00	683	1.00	671	1.00
	-28 to -1 days	183	0.60 (0.51, 0.71)			183	0.59 (0.49, 0.70)	181	0.62 (0.52, 0.74)	183	0.59 (0.50, 0.70)	167	0.58 (0.49, 0.70)
	0 day	*	0.08 (0.01, 0.57)	*	0.06 (0.01, 0.45)	*	0.08 (0.01, 0.55)	*	0.08 (0.01, 0.59)	*	0.08 (0.01, 0.56)	*	0.09 (0.01, 0.61)
	1-7 days	89	1.01 (0.80, 1.27)	93	0.85 (0.60, 1.20)	93	1.02 (0.82, 1.28)	93	1.09 (0.87, 1.37)	93	1.03 (0.82, 1.29)	76	0.91 (0.71, 1.16)
	8-14 days	85	0.94 (0.75, 1.19)	88	0.86 (0.63, 1.17)	88	0.94 (0.75, 1.18)	86	0.99 (0.78, 1.26)	88	0.95 (0.75, 1.19)	71	0.83 (0.65, 1.07)
	15-21 days	103	1.16 (0.93, 1.43)	107	1.11 (0.84, 1.45)	107	1.16 (0.94, 1.43)	96	1.21 (0.96, 1.52)	107	1.16 (0.94, 1.44)	91	1.09 (0.87, 1.37)
	22-28 days	97	1.13 (0.91, 1.41)	102	1.12 (0.87, 1.43)	102	1.14 (0.92, 1.41)	83	1.16 (0.91, 1.47)	100	1.15 (0.93, 1.42)	97	1.19 (0.96, 1.49)
**SARS-CoV-2 positive test**	baseline	133	1.00	148	1.00					132	1.00		
	-28 to -1 days	162	4.81 (3.75, 6.18)							164	4.71 (3.68, 6.02)		
	0 day	55	36.71 (26.46, 50.93)	90	46.11 (32.53, 65.37)					55	35.17 (25.42, 48.67)		
	1-7 days	57	5.30 (3.84, 7.31)	66	4.86 (3.38, 6.98)					59	5.35 (3.91, 7.33)		
	8-14 days	63	5.66 (4.15, 7.72)	75	5.49 (3.91, 7.71)					64	5.62 (4.14, 7.62)		
	15-21 days	34	3.09 (2.11, 4.54)	45	3.30 (2.27, 4.80)					34	2.98 (2.03, 4.36)		
	22-28 days	31	2.76 (1.86, 4.10)	37	2.70 (1.82, 3.99)					31	2.66 (1.79, 3.95)		

Supplementary table 7c: Incidence rate ratios (IRR 95% CI) for coeliac (negative control) and anaphylaxis (positive control) in pre-defined risk periods immediately before and after exposure to vaccination, adjusted for calendar time from December 1, 2020 to April 24, 2021. Comparisons between different sensitivity analyses. (see footnote table 5a for description of sensitivity analyses)

		Sensitivity 1*		Sensitivity 2*		Sensitivity 3*		Sensitivity 4*		Sensitivity 5*		Sensitivity 6*	
		events	IRR (95% CI)	events	IRR (95% CI)	events	IRR (95% CI)	events	IRR (95% CI)	events	IRR (95% CI)	events	IRR (95% CI)
**Coeliac**													
**ChAdOx1nCoV-19 vaccine**	baseline	2070	1.00	516	1.00	2072	1.00	1555	1.00	1881	1.00	2072	1.00
	-28 to -1 days	688	0.95 (0.87, 1.05)			688	0.95 (0.87, 1.05)	614	0.92 (0.83, 1.02)	678	0.95 (0.87, 1.05)	688	0.96 (0.87, 1.05)
	0 day	*	0.08 (0.02, 0.31)	*	0.10 (0.02, 0.40)	*	0.08 (0.02, 0.31)	*	0.10 (0.03, 0.42)	*	0.08 (0.02, 0.31)	*	0.08 (0.02, 0.31)
	1-7 days	157	0.88 (0.74, 1.04)	157	1.11 (0.86, 1.43)	157	0.88 (0.74, 1.04)	108	0.85 (0.69, 1.05)	154	0.88 (0.74, 1.04)	157	0.88 (0.75, 1.04)
	8-14 days	180	1.03 (0.88, 1.21)	180	1.27 (1.00, 1.59)	180	1.03 (0.88, 1.21)	114	1.08 (0.88, 1.34)	168	1.04 (0.88, 1.21)	180	1.04 (0.89, 1.22)
	15-21 days	158	0.96 (0.81, 1.14)	158	1.15 (0.93, 1.44)	158	0.96 (0.81, 1.14)	89	0.98 (0.78, 1.24)	141	0.97 (0.82, 1.14)	158	0.97 (0.82, 1.14)
	22-28 days	152	1.00 (0.85, 1.19)	152	1.18 (0.96, 1.44)	152	1.00 (0.84, 1.19)	72	0.97 (0.75, 1.25)	117	1.02 (0.86, 1.20)	152	1.01 (0.85, 1.20)
**BNT162b2 mRNA vaccine**	baseline	1277	1.00	607	1.00	1277	1.00	887	1.00	1167	1.00	1213	1.00
	-28 to -1 days	428	0.88 (0.78, 0.99)			428	0.87 (0.78, 0.98)	422	0.88 (0.78, 1.00)	424	0.88 (0.78, 0.98)	391	0.89 (0.79, 1.01)
	0 day	*	0.22 (0.08, 0.58)	*	0.28 (0.10, 0.79)	*	0.22 (0.08, 0.58)	*	0.22 (0.08, 0.60)	*	0.22 (0.08, 0.58)	*	0.24 (0.09, 0.65)
	1-7 days	95	0.74 (0.60, 0.92)	95	0.94 (0.69, 1.28)	95	0.74 (0.60, 0.92)	93	0.74 (0.60, 0.92)	94	0.74 (0.60, 0.92)	83	0.72 (0.57, 0.90)
	8-14 days	121	0.94 (0.77, 1.13)	121	1.11 (0.85, 1.45)	121	0.93 (0.77, 1.13)	107	0.88 (0.71, 1.08)	121	0.93 (0.77, 1.13)	110	0.94 (0.77, 1.15)
	15-21 days	116	0.89 (0.73, 1.08)	118	1.06 (0.83, 1.35)	118	0.90 (0.74, 1.09)	95	0.95 (0.76, 1.18)	117	0.90 (0.74, 1.09)	110	0.94 (0.77, 1.15)
	22-28 days	107	0.85 (0.70, 1.04)	107	0.95 (0.75, 1.20)	107	0.85 (0.69, 1.03)	66	0.85 (0.66, 1.10)	107	0.85 (0.69, 1.04)	102	0.88 (0.71, 1.08)

**SARS-CoV-2 positive test**	baseline	173	1.00							160	1.00		
	-28 to -1 days	83	1.98 (1.50, 2.61)							83	1.98 (1.51, 2.61)		
	0 day	41	25.93 (18.26, 36.83)							39	26.01 (18.31, 36.95)		
	1-7 days	66	5.85 (4.36, 7.86)							66	5.88 (4.38, 7.90)		
	8-14 days	51	4.29 (3.11, 5.92)							51	4.30 (3.12, 5.93)		
	15-21 days	15	1.19 (0.70, 2.03)							15	1.19 (0.70, 2.03)		
	22-28 days	19	1.40 (0.87, 2.25)							19	1.40 (0.87, 2.26)		
**Anaphylaxis**													
**ChAdOx1nCoV-19 vaccine**	baseline	339	1.00			339	1.00	228	1.00	307	1.00	339	1.00
	-28 to -1 days	105	0.88 (0.69, 1.11)			105	0.87 (0.69, 1.10)	93	0.91 (0.70, 1.20)	104	0.88 (0.69, 1.11)	105	0.86 (0.68, 1.09)
	0 day	45	10.75 (7.75, 14.92)			45	10.68 (7.70, 14.81)	26	9.03 (5.77, 14.13)	43	10.73 (7.74, 14.89)	45	10.57 (7.62, 14.66)
	1-7 days	49	1.72 (1.25, 2.36)			49	1.70 (1.24, 2.34)	31	1.61 (1.05, 2.46)	47	1.71 (1.24, 2.34)	49	1.69 (1.23, 2.32)
	8-14 days	23	0.83 (0.54, 1.28)			23	0.82 (0.53, 1.27)	17	1.00 (0.58, 1.71)	21	0.83 (0.54, 1.28)	23	0.81 (0.53, 1.26)
	15-21 days	28	1.05 (0.70, 1.57)			28	1.04 (0.69, 1.55)	11	0.72 (0.38, 1.39)	23	1.05 (0.70, 1.57)	28	1.03 (0.69, 1.54)
	22-28 days	23	0.96 (0.62, 1.49)			23	0.95 (0.61, 1.47)	13	1.08 (0.58, 2.00)	19	0.97 (0.63, 1.51)	23	0.94 (0.61, 1.46)
**BNT162b2 mRNA vaccine**	baseline	108	1.00			109	1.00	70	1.00	98	1.00	105	1.00
	-28 to -1 days	30	0.75 (0.49, 1.13)			30	0.73 (0.48, 1.10)	27	0.67 (0.42, 1.05)	29	0.73 (0.48, 1.11)	29	0.73 (0.48, 1.11)
	0 day	29	19.29 (12.64, 29.44)			29	18.64 (12.22, 28.42)	28	18.62 (11.81, 29.35)	28	18.87 (12.38, 28.78)	26	17.59 (11.27, 27.46)
	1-7 days	16	1.51			16	1.46	15	1.44	15	1.48	14	1.34

			(0.88, 2.58)				(0.86, 2.50)		(0.81, 2.55)		(0.87, 2.53)		(0.76, 2.37)
	8-14 days	21	1.93 (1.19, 3.13)			21	1.87 (1.16, 3.02)	20	1.93 (1.15, 3.24)	21	1.89 (1.17, 3.06)	19	1.78 (1.07, 2.95)
	15-21 days	12	1.14 (0.62, 2.08)			12	1.10 (0.60, 2.01)	9	1.03 (0.50, 2.10)	12	1.11 (0.61, 2.03)	11	1.06 (0.56, 2.00)
	22-28 days	10	0.94 (0.49, 1.82)			10	0.91 (0.47, 1.76)	6	0.78 (0.33, 1.85)	10	0.93 (0.48, 1.78)	10	0.96 (0.50, 1.85)
**SARS-CoV-2 positive test**	baseline	43	1.00							40	1.00		
	-28 to -1 days	14	1.55 (0.81, 2.97)							14	1.56 (0.81, 2.97)		
	0 day	9	25.83 (12.17, 54.84)							9	25.67 (12.10, 54.46)		
	1-7 days	8	3.23 (1.48, 7.06)							8	3.21 (1.47, 7.02)		
	8-14 days	*	0.41 (0.06, 2.98)							*	0.41 (0.06, 2.97)		
	15-21 days	*	0.38 (0.05, 2.78)							*	0.38 (0.05, 2.77)		
	22-28 days	5	1.74 (0.68, 4.45)							5	1.73 (0.68, 4.43)		

Supplementary table 8: Measures of effect of vaccinations and SARS-CoV-2 infections presented in absolute terms, number needed to expose for one excess event; excess events due to exposure; excess events per 10 million exposed. Only associations significant at the 0.01 level were reported over the 8-28 days post exposure. When IRR were not significant over the 8-28 days post-vaccine, absolute measures were not given.

	IRR (95% CI) 8-28 days*	Number needed to expose to cause one excess event (95% CI)	Excess events per 10,000,000 exposed (95% CI)
**Thrombocytopenia**			
with ChAdOx1nCoV-19 vaccine	1.23 (1.14, 1.33)	92714 (69872, 141171)	107 (71, 143)
with BNT162b2 mRNA vaccine	1.06 (0.97, 1.16)	*	*
with SARS-CoV-2 infection	2.85 (2.42, 3.35)	10710 (9910, 11848)	934 (844, 1009)
**Venous thromboembolism**			
with ChAdOx1nCoV-19 vaccine	1.06 (1.01, 1.12)	152066 (80337, 869363)	66 (11, 124)
with BNT162b2 mRNA vaccine	0.91 (0.83, 1.01)	*	*
with SARS-CoV-2 infection	8.06 (7.49, 8.66)	792 (785, 801)	12614 (12479, 12738)
**Arterial thrombosis**			
with ChAdOx1nCoV-19 vaccine	1.02 (0.99, 1.05)	*	*
with BNT162b2 mRNA vaccine	1.00 (0.97, 1.03)	*	*
with SARS-CoV-2 infection	2.53 (2.38, 2.70)	1794 (1723, 1871)	5573 (5344, 5802)
**CVST**			
with ChAdOx1nCoV-19 vaccine	2.37 (1.34, 4.21)	1356817 (1028657, 3091145)	7 (3, 10)
with BNT162b2 mRNA vaccine	1.93 (0.87, 4.28)	*	*

with SARS-CoV-2 infection	7.81 (1.64, 37.10)	504297 (451907, 1126798)	20 (9, 22)
**Ischaemic stroke**			
with ChAdOx1nCoV-19 vaccine	1.04 (0.99, 1.08)	*	*
with BNT162b2 mRNA vaccine	1.06 (1.01, 1.12)	69567 (36752, 397713)	143 (25, 272)
with SARS-CoV-2 infection	2.14 (1.93, 2.38)	5885 (5407, 6506)	1699 (1536, 1849)
**Myocardial infarction**			
with ChAdOx1nCoV-19 vaccine	1.01 (0.98, 1.04)	*	*
with BNT162b2 mRNA vaccine	0.98 (0.95, 1.02)	*	*
with SARS-CoV-2 infection	2.58 (2.39, 2.80)	2752 (2622, 2898)	3633 (3450, 3814)
**Other rare arterial thrombotic event**			
with ChAdOx1nCoV-19 vaccine	1.08 (0.95, 1.22)	*	*
with BNT162b2 mRNA vaccine	1.08 (0.94, 1.25)	*	*
with SARS-CoV-2 infection	3.72 (2.90, 4.77)	18504 (17119, 20651)	540 (484, 584)
